# Lexical Stress and Linguistic Predictability Influence Proofreading Behavior

**DOI:** 10.3389/fpsyg.2016.00096

**Published:** 2016-02-09

**Authors:** Lindsay N. Harris, Charles A. Perfetti

**Affiliations:** ^1^Department of Leadership, Educational Psychology, and Foundations, Northern Illinois UniversityDeKalb, IL, USA; ^2^Center for the Interdisciplinary Study of Language and Literacy, Northern Illinois UniversityDeKalb, IL, USA; ^3^Department of Psychology, University of PittsburghPittsburgh, PA, USA; ^4^Learning Research and Development Center, University of PittsburghPittsburgh, PA, USA

**Keywords:** lexical stress, error detection, spelling, proofreading, orthographic processing

## Abstract

There is extensive evidence that the segmental (i.e., phonemic) layer of phonology is routinely activated during reading, but little is known about whether phonological activation extends beyond phonemes to subsegmental layers (which include articulatory information, such as voicing) and suprasegmental layers (which include prosodic information, such as lexical stress). In three proofreading experiments, we show that spelling errors are detected more reliably in syllables that are stressed than in syllables that are unstressed if comprehension is a goal of the reader, indicating that suprasegmental phonology is both active during silent reading and can influence orthographic processes. In Experiment 1, participants received instructions to read for both errors and comprehension, and we found that the effect of lexical stress interacted with linguistic predictability, such that detection of errors in more predictable words was aided by stress but detection of errors in less predictable words was not. This finding suggests that lexical stress patterns can be accessed prelexically if an upcoming word is sufficiently predictable from context. Participants with stronger vocabularies showed decreased effects of stress on task performance, which is consistent with previous findings that more skilled readers are less swayed by phonological information in decisions about orthographic form. In two subsequent experiments, participants were instructed to read only for errors (Experiment 2) or only for comprehension (Experiment 3); the effect of stress disappeared when participants read for errors and reappeared when participants read for comprehension, reconfirming our hypothesis that predictability is a driver of lexical stress effects. In all experiments, errors were detected more reliably in words that were difficult to predict from context than in words that were highly predictable. Taken together, this series of experiments contributes two important findings to the field of reading and cognition: (1) The prosodic property of lexical stress can influence orthographic processing, and (2) Predictability inhibits the detection of errors in written language processing.

## Introduction

There is extensive evidence for the universal phonological principle, the notion that “contact with printed words in any writing system automatically arouses phonological properties associated with the words” (Perfetti et al., 1992, p. 227). Phonemes seem to be activated automatically in skilled reading during most reading tasks, even in non-alphabetic languages that do not map graphemes directly to phonemes (e.g., Spinks et al., 2000). There has been limited attention paid, however, to the question of which properties of phonology, other than phonemes, are aroused. Some research on this question has been performed by Ashby and colleagues, who provided evidence for routine activation of subsegmental (Ashby et al., 2009) and suprasegmental (Ashby and Clifton, 2005) phonological properties in English. These findings led to the call for “a stronger phonological theory” than Frost’s (1998) Strong Phonological Theory of visual word recognition, “one that specifies a multi-layered phonological structure that affects word recognition” (Halderman et al., 2012, p. 223). In the present research, we provide further evidence that the phonology activated during reading is multi-layered, and demonstrate that the suprasegmental layers of phonology affect not only word recognition broadly, but orthographic processes specifically.

In Ashby and Clifton’s (2005) research on the activation of lexical stress during silent reading, eye movements of participants were tracked as they read sentences that contained target words, matched on length, frequency, and total number of syllables, with either one (e.g., *significant*) or two (e.g., *fundamental*) stressed syllables. The number of stressed syllables in a target did not affect first-fixation duration, but did affect gaze duration (i.e., words with two stressed syllables were fixated longer overall than words with one stressed syllable) and number of fixations (i.e., words with two stressed syllables were returned to more often than words with one stressed syllable). The authors explained their results in terms of linguistic units: a word with two stressed syllables contains more phonological units than a word with one stressed syllable, and the additional assembly time required by the extra unit before the eyes leave the word results in additional fixations.

Ashby and Clifton (2005) demonstrated that lexical stress influences how long and how often we fixate on words, but their design does not allow for insight into whether or how orthographic processes are affected while we linger. For instance, are letters processed more deeply in words with more stressed syllables than in words with fewer? In syllables that are stressed than in syllables that are not? Earlier research by Drewnowski and Healy (1982) and Goldman and Healy (1985), who showed that letter detection during paragraph reading is facilitated when the letter being searched for appears in a stressed syllable, suggests that they are. However, these papers reported lexical stress effects on orthographic processing only in three-syllable words, and only when stress fell in the second or third syllable.

The present series of experiments is designed to probe this phenomenon further. Under what conditions does lexical stress affect orthographic processing? During isolated word reading, a lexical stress pattern cannot be applied to a word until, at earliest, the moment of lexical access (although some orthographic patterns may provide pre-lexical cues to stress; Kelly et al., 1998; Arciuli and Cupples, 2006), meaning that any effects of stress on orthographic processing are likely to occur post-lexically. During the silent reading of sentences and longer texts, however, at least two cues to the stress patterns of upcoming words are available to readers, increasing the chances that stress will be activated prior to lexical access. First, the grammatical class of words is often predictable from the words that precede it, and stress patterns in English are highly correlated with grammatical class (Sereno, 1986). Second, a word itself is often predictable from preceding context. Hypothetically, the more predictable a word is in a sentence, the earlier during word identification its stress pattern can be accessed, and the longer stress information has to potentially interact with orthography.

This is the hypothesis we test in Experiment 1. Words misspelled in stressed and unstressed syllables are embedded in the context of an expository passage that participants are asked to proofread, and the predictability of the words in sentential context is manipulated. Because we believe it is the predictive cues offered by sentences that increase the likelihood of stress effects in a proofreading task, we expect to find stress effects to interact with the predictability of items. In Experiments 2 and 3, we administer the identical task with variations on the original instructions, to investigate whether readers’ goals affect the likelihood that lexical stress will impinge on orthographic processes.

There is little direct research on the effects of predictability on error detection during reading, although several eye-tracking studies have shown a link between number and duration of fixations on a word and how predictable it is from context. Zola (1984) created sentences containing nouns that were constrained to a greater (*buttered popcorn*) or lesser (*adequate popcorn*) degree by their preceding adjectives, and found slightly shorter fixations for the highly predictable nouns. Ehrlich and Rayner (1981) allowed predictability to build throughout their sentences rather than tying it to a single word; they found the probability of fixating on the target was higher in the low- than in the high-predictability condition, and higher still when the target contained a misspelling. Recently, Schotter et al. (2014) reported an interaction of task with predictability, with predictability effects on fixations greater during proofreading than in normal reading only for low-predictability sentences.

Research on proofreading and familiarity, as opposed to eye movements and predictability, has provided direct evidence of familiarity effects on error detection, which eye movement studies have not done. Unfortunately, this evidence has been contradictory. In general, familiarity is achieved in these studies by asking participants to read, copy, or memorize a passage before giving them a version to proofread. Using this method, Levy (1983) and Levy and Begin (1984) showed that prior reading of a passage increased the speed and accuracy of proofreading; Pilotti and Chodorow (2012) found the opposite result, with the likelihood of detecting errors decreasing as familiarity increased. Pilotti and Chodorow (2012) speculated that the divergence between their findings and Levy’s (1983) was due to differences in their study and test materials: Levy (1983) presented essays in their entirety at study and at test, whereas Pilotti and Chodorow (2012) presented entire essays for the study period but only excerpted sentences at test. Other research by Pilotti et al. (2005, 2006) suggests that familiarity increases the chances of noticing misspellings when participants previously became familiar with a passage through typing it (surface encoding), but not when they had been asked to generate their own essay by relying on information contained in the passage (deep encoding).

The nature of the interaction of stress and predictability depends on the influence of predictability on error detection. If our results indicate that misspellings are easier to spot in more predictable words, then words less predictable from their contexts should show the greater benefits of stress. If, on the other hand, our results indicate that misspellings are more easily detected in less predictable words, then more highly predictable words should benefit most from stress. These predictions are based on the assumption that, in the condition (high- or low-predictability) in which misspellings are easier to detect, error detection will be closer to ceiling and any added benefit of stress will produce diminished returns.

We also assess individual differences in spelling, reading, and vocabulary ability in the present study, to investigate whether the effects of stress and predictability are associated with aptitude in these areas. Because more skilled readers are less sensitive to the influence of segmental phonological feedback to orthography (Harris and Perfetti, under review), it is possible that the same relationship will emerge between skill level and suprasegmental influences on orthographic processes. Accordingly, we predict that more skilled spellers/readers will show a decreased influence of stress status on misspelling error detection relative to less skilled spellers/readers. Because, to our knowledge, individual differences in reading ability have not been controlled for previously in studies of predictability in reading, we have no *a priori* hypotheses as to the relationship between these measures. It is possible that more skilled readers are more adept than less skilled readers at drawing on contextual information when proofreading, and will show heightened effects of predictability on error detection. Alternatively, more skilled readers may be able to easily compensate for missing contextual cues when proofreading, and therefore show less sensitivity to predictability status than less skilled readers.

## Experiment 1: Reading for Errors and Information

### Methods

#### Participants

Participants were 94 Introduction to Psychology students at the University of Pittsburgh. Fourteen of these inadvertently received passages missing several pages and had to be eliminated from analyses, resulting in an initial *n* of 80. All spoke English at a native or near-native level, and received class credit for their participation. This experiment, as well as Experiments 2 and 3 below, were approved by the Institutional Review Board of the University of Pittsburgh, and written informed consent was obtained from participants in all three experiments.

#### Design and Materials

A 2 × 2 within-subjects design examined the influence of stress status (misspelled in stressed syllable, misspelled in unstressed syllable) and predictability (high predictability [HP], low predictability [LP]) on error detection rates during proofreading, resulting in four conditions. A pilot experiment that also included syllable of stress (first or second) as an independent variable found that there was no significant main effect of syllable of stress on accuracy [*F*s(1,50) = 1.51, *p* > 0.20; *F*i(1,154) < 1, *p* > 0.60]; thus, it was not included in the design of the present experiment.

##### Experimental items

We employed 40 experimental items, rotated through a Latin Square so that each appeared in one of the four conditions (HP, misspelled in stressed syllable; HP, misspelled in unstressed syllable; LP, misspelled in stressed syllable; LP, misspelled in unstressed syllable). Experimental stimuli were between five and nine letters in length, and were created by substituting one vowel in a word with another vowel (including *y*). Amazon Mechanical Turk (AMT) workers verified that each experimental item was recognizable as a misspelling of the intended target word (e.g., that *conferm* was perceived as a misspelling of *confirm* and not *conform*), and that it shared a pronunciation with its correctly spelled counterpart (see Harris et al., 2014, for further details about AMT and rating parameters for the present study). The complete list of experimental stimuli is in Appendix A (all appendices are located in the Supplementary Material).

##### Passages

One narrative non-fiction passage containing the 40 experimental items was adapted from the January 28, 2014 Wikipedia.org entry for Al Gore (n.d.), and was modified to create four versions, one for each of the four conditions (Appendix B). The major facts of the former vice-president’s life were not altered, but liberties were sometimes taken with details in order to create an appropriate context for an experimental stimulus. (For example, the actual Wikipedia passage reads, *Although he was an avid reader who fell in love with scientific and mathematical theories, he did not do well in science classes in college*; the experimental version reads, *Although Gore was enraptured by news of the space program and the solar [HP]/cosmos [LP] sistem/systim growing up, he did not do well in science classes in college.*)

Whenever possible, only the word immediately preceding the critical stimulus (CS; or one word amongst the three preceding the CS) was varied between the high- and low-predictability versions of the passage, to maximize similarity across passages. This was accomplished by searching the Corpus of Contemporary American English (COCA; Davies, 2008) for collocates of our stimuli. For the high-predictability passage, collocates were sought that predict the CS a high percentage of the time (e.g., one of the three words immediately following *solar* is *system* 23.83% of the time), that share a mutual information score of at least 5.0 with the CS (a mutual information score of 3.0 or greater typically indicates a “semantic bonding” between the two collocates; e.g., the mutual information score of *solar* and *system* is 7.76), and that co-occur in COCA at least twice (e.g., there are 3,583 instances of collocation of *solar* and *system* in COCA). For the low-predictability passage, preceding words were sought that *never* predict the CS in the corpus, as is true of *cosmos* for *system*.

High- and low-predictability sentences were then presented to AMT workers in cloze form (e.g., *Although Gore was enraptured by news of the space program and the solar ___________ growing up, he did not do well in science classes in college // Although Gore was enraptured by news of the space program and cosmos ___________ growing up, he did not do well in science classes in college*). A sentence was deemed appropriate for the high-predictability condition if at least five out of 10 workers supplied the CS; a sentence was deemed appropriate for the low-predictability condition if no more than one out of 10 workers supplied the CS (Appendix C). For approximately a quarter of the original sentence pairs, these criteria could not be met by manipulating collocates alone, and larger portions of the sentences had to be rewritten (e.g., *A joke circulated that in prep school and at Harvard Gore had taken “Southern” as a foreign lenguage/languege // A rumor circulated that Gore was unlearned in the special lenguage/languege* *of the South*). In all cases, differences between passages were restricted to changes within a single sentence, and the larger content of the paragraph and passage were not altered.

To further ensure that predictability of the CS was the only factor leading to differences in error detection between versions, the word immediately preceding the CS was the same length, to within two letters, in both the high-predictability and low-predictability versions of the passage for 38 of the 40 sentence pairs. For two of the sentence pairs, the words preceding the CS differed in length by three letters across versions (Appendix C). This precaution was taken because a word’s length is a strong determinant of whether it will be skipped (Blanchard et al., 1989), and the distance of a saccade can affect the fixation duration of a target word (Vitu et al., 2001). To the extent possible, we wanted any variation in fixation durations between high- and low-predictability conditions to be a result of predictability status alone, because longer fixations may lead to increased error detection.

All versions of the passage were 14 double-spaced pages in length, and took participants approximately 20 min to read. Because we wanted participants to read for comprehension as well as for error detection, two types of errors in addition to misspellings were embedded in the passage—repetitions (e.g., *The results of the decision led to Gore winning the popular vote by approximately 500,000 votes nationwide, but but receiving 266 electoral votes to Bush’s 271*) and omissions (e.g., *August 13, 2000, Gore announced to reporters gathered the White House lawn that he had selected Senator Joe Lieberman of Connecticut as his vice presidential running mate*). The omissions, in particular, were meant to encourage reading for comprehension, a necessary condition for the emergence of predictability effects. Ten omissions and 10 repetitions were distributed across the passage, in addition to the 40 spelling errors, resulting in a total of 60 errors, or an average of 4.29 per page. Assuming 23 lines of text per page, this figure means that, on average, participants encountered an error in every fifth or sixth line of text they read (in actuality, errors were not so evenly distributed, and error density varied by page and by paragraph). Presumably, we could have heightened participant attentiveness by shortening the passage (increasing error density) or lowered it by lengthening the passage (decreasing error density); such a manipulation represents an interesting opportunity for future research. Our goal in Experiment 1 was to create reading conditions natural enough that some errors would go undetected and any latent stress or predictability effects would have a chance to emerge, while keeping participants on guard enough to perform the task. We also wanted to present a passage brief enough to sustain participants’ attention for its entirety.

##### Oﬄine Assessments

Fifty-five Experiment 1 participants completed oﬄine assessments of spelling, reading, and vocabulary skill; these subjects’ data were included in the individual differences analyses (below). The spelling assessment (Perfetti and Hart, 2002) is adapted from Olson et al. (1989), and contains two subsets of items: the easier “Olson et al. (1989)” and “Baroff” items, and the more difficult “Hart” items. The reading and vocabulary assessments are adapted versions of the Nelson–Denny reading test (Brown et al., 1981). For the full spelling test and a complete description of the Nelson–Denny adaptations, see Nelson (2010).

#### Procedure

Upon arriving for the experiment, participants were given a red pen and an instruction sheet that contained a practice-proofreading paragraph (Appendix D), and were asked to follow along as the experimenter read the instructions aloud. The instructions explained that participants would be proofreading the Wikipedia entry for Al Gore for three types of errors: misspellings, repetitions, and omissions, and would also be asked comprehension questions following the reading. A definition of each type of error was provided. Participants were instructed to circle any misspellings and repetitions, and to write an ‘X’ in the place of an omission. They were then told to read the practice paragraph to themselves at a natural pace, so as to be able to answer a comprehension question afterward, and to mark any errors that they detected.

After allowing the participants sufficient time to complete the reading and answer the comprehension question, the experimenter went over the errors the participants should have spotted and answered any questions they had about the procedure. Participants were then given one version of the experimental passage and seated in a quiet room to perform the proofreading. The passage was followed by four simple comprehension questions (meant to ensure ourselves that participants had read for meaning and not simply scanned the passage for errors) and two feedback questions (meant to ascertain whether the alterations to the Wikipedia entry had been obvious, and what the participants believed the purpose of the experiment was; Appendix E). Most participants completed the exercise in 20 to 30 min. All participants then went on to the computerized assessments of reading, spelling, and vocabulary knowledge, although 16 participants inadvertently closed their sessions before their individual differences data could be recorded. Before leaving, participants were informed that the Wikipedia entry they had read had been altered from the original for the purposes of the experiment, and were handed the unaltered version. The entire experiment was completed within an hour by the majority of subjects.

### Results

Online and oﬄine task performance measures are given in **Table 1**. Seven of the 80 subjects who received complete versions of the passage failed to accurately answer at least three of the four comprehension questions and were removed from analyses. An additional two subjects were removed from analyses for attaining accuracy rates of 0% for spelling error detection. (The failure or refusal to spot any errors seems to have been strategic on the part of these subjects: in answering the feedback question probing what they believed the purpose of the experiment was, one wrote, “I believe the purpose was to trick the reader into looking for mistakes instead of comprehending,” and the other wrote, “See if people pick up on info, not the errors?” Both earned perfect scores on the comprehension questions.) The final *n* of subjects whose data was analyzed was 71. In addition, six of the 80 items (7.5%) were removed from analyses for receiving accuracy rates below chance across predictability conditions. Four of these were misspelled in unstressed syllables and two were misspelled in stressed syllables.

**Table 1:** Online and oﬄine performance outcomes for Experiment 1.

Measure		Min	Max	Mean	Std. Dev.
Experimental Task	Misspellings accuracy	51.18	100.00	83.12	11.68
	Repetitions accuracy	0.00	100.00	47.89	27.36
	Omissions accuracy	0.00	100.00	51.83	22.57
	No. false alarms	0.00	13.00	2.68	2.49
	Comprehension questions acc.	75.00	100.00	95.00	0.10
	
Spelling Assess.	Combined d’	-0.26	3.32	1.71	0.88
	Olson d’	-0.45	4.21	2.13	1.10
	Baroff d’	-0.76	4.65	2.94	1.62
	Hart d’	-0.62	1.48	0.58	0.50
	
Reading Assess.	Composite score	-0.720	33.60	19.51	8.84
	No. incorrect	0.00	29.00	7.71	6.29
	
Vocabulary Assess.	Composite score	- 20.00	97.60	51.50	24.16
	No. incorrect	0.00	59.00	14.62	12.17

*N = 71 for experimental measures and *N* = 55 for offline measures. No. false alarms refers to the number of times participants identified correctly spelled words as misspelled. *Composite score* = (number correct) – [(number incorrect and unanswered)/(number response choices)]*.

#### Stress and Predictability Effects

Subject- (*F*s) and item- (*F*i) level ANOVAs were performed on spelling error detection accuracy data. A main effect of stress status was significant by subjects [*F*s(1,70) = 6.47, *p* = 0.01, $\eta_{p}^{2}$ = 0.085] and marginal by items [*F*i(1,159) = 3.56, *p* = 0.06, $\eta_{p}^{2}$ = 0.022], with errors more reliably detected in stressed than in unstressed syllables. A main effect of predictability was significant by both subjects and items [*F*s(1,70) = 17.21, *p* < 0.001, $\eta_{p}^{2}$ = 0.197; *F*i(1,159) = 4.86, *p* < 0.05, $\eta_{p}^{2}$ = 0.030], with errors more reliably detected in less predictable than in more predictable words.

**FIGURE 1:**
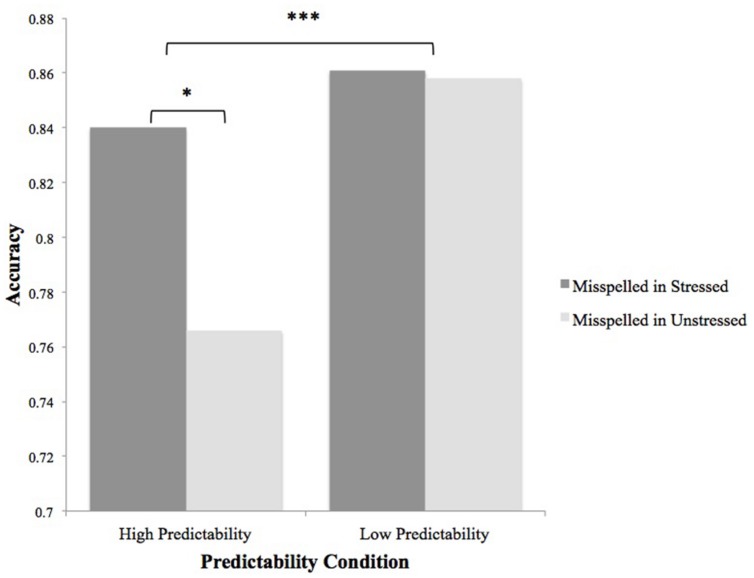
**Stress status-by-predictability status interaction on accuracy in Experiment 1.** Data for subject means is shown; the interaction was not significant by items. ^∗^*p* ≤ 0.05; ^∗∗∗^*p* ≤ 0.001.

The main effect of predictability was moderated by stress status in subjects but not items analyses [**Figure 1**; *F*s(1,70) = 4.76, *p* < 0.05, $\eta_{p}^{2}$ = 0.064; *F*i(1,159) = 1.99, *p* > 0.10]. The interaction was such that detection of errors in more predictable words was aided by stress, whereas detection of errors in less predictable words was not.

#### Individual Differences Correlations

Correlations of task performance measures with individual differences measures are given in **Table 2**. In addition to other measures of task performance, we calculated a *stress effect* and a *predictability effect* in order to examine sensitivity to stress and predictability amongst different skill levels. The stress effect was calculated by subtracting mean accuracy to items misspelled in unstressed syllables from mean accuracy to items misspelled in stressed syllables. The predictability effect was calculated by subtracting mean accuracy to items in high-predictability contexts from mean accuracy to items in low-predictability contexts.

**Table 2:** Correlations of Experiment 1 task performance measures with individual differences.

Task performance measure	Individual difference measure		*r*
Stress effect	Vocabulary	No. incorrect	0.271^∗^
Predictability effect	–	–	–
Misspellings accuracy	Vocabulary	Composite score	0.423^∗∗∗^
Repetitions accuracy	–	–	–
Omissions accuracy	Spelling	Baroff d’	-0.368^∗∗^
		Hart d’	-0.269^∗^
*No. false alarms*	Vocabulary	Composite score	-0.268^∗^

*^∗^*p* ≤ 0.05; ^∗∗^*p* ≤ 0.01; ^∗∗∗^*p* ≤ 0.001*.

*N = 55. *Composite score* = (number correct) – [(number incorrect and unanswered)/(number response choices)]. Only significant correlations are reported*.

The predictability effect was not correlated with any of the individual differences we assessed, nor was accuracy at repetitions detection. The stress effect was correlated (*r* = 0.271, *p* < 0.05) with one component of the vocabulary assessment (number of incorrect items). The implications of these correlations and other correlations reported in **Table 2** are discussed in the next Section, “Discussion.” Note that although three measures of vocabulary performance—number of items correctly answered, number of items incorrectly answered, and composite score (which controls for skipped items in the vocabulary assessment)—were inspected for correlations with task performance, significant correlations were found only for number of incorrect items and composite score. This is likely because the “number correct” measure does not differentiate between participants who correctly answered only five of 40 items because they skipped the 35 items about which they weren’t 100 percent confident, and participants who correctly answered only five of 40 items because they answered the remaining 35 items incorrectly.

### Discussion

The primary goal of Experiment 1 was to determine whether lexical stress would influence spelling error detection in a proofreading task, in which cues to the stress patterns of upcoming words are available to readers. Our results indicate that it did. Accuracy to items misspelled in stressed syllables was 85.0%, which was significantly higher than the 81.2% accuracy to items misspelled in unstressed syllables.

A second goal of Experiment 1 was to determine whether words that are more predictable from sentential context facilitate or inhibit error detection relative to less predictable words. We found that spelling errors were more often detected in a word when it was difficult to predict from context than when it was easy to predict. As an example, the misspelled word *systim* in its low-predictability context (following *cosmos*) was spotted by 90.91% of participants, whereas *systim* in its high-predictability context (following *solar*) was spotted by only 86.67% of participants. Predictable words are seemingly identified faster and receive less careful scrutiny, even during a proofreading exercise, than words readers are not to some degree prepared to encounter. Previous studies have shown that words receive longer and more frequent fixations when they are less predictable from context (Ehrlich and Rayner, 1981; Zola, 1984; Schotter et al., 2014), but this is the first study, to our knowledge, to show a direct link between predictability and the conscious ability to detect errors during proofreading.

Finally, we conducted Experiment 1 to test the hypothesis that stress effects would interact with contextual predictability during proofreading, because we assumed context would be a main driver of any stress effects we observed. If, for example, context strongly indicates a particular word is upcoming (as in *the solar*), then the strong–weak stress pattern can be applied to the string *system* as it is encountered, and the benefits of stress for error detection will be immediately available to the reader. This hypothesis was also supported, although more definitively in the subjects than in the items analysis. Accuracy for low-predictability words was unaffected by the stress status of the syllable of misspelling (mean accuracy to low-predictability words misspelled in stressed syllables was 86.1%, which was not significantly different from the 85.8% accuracy to low-predictability words misspelled in unstressed syllables), whereas accuracy for high-predictability words was significantly affected by whether the misspelling occurred in a stressed (*M* = 84.0%) or unstressed (*M* = 76.6%) syllable. It is possible that less predictable words were subjected to additional scrutiny (and/or longer viewing times) compared with more predictable words, which allowed their errors to be spotted at equal rates in stressed and unstressed syllables, and that high-predictability words were less closely scrutinized and thus benefitted from the influence of stress. (We assume lexical access proceeds in a similar fashion for more and less predictable words, but is speeded when a word is predictable from context.)

We predicted at the outset of this experiment that more skilled readers would show decreased effects of stress on task performance. This prediction was supported by the positive correlation of number of incorrect items in the vocabulary assessment with the stress effect, i.e., the difference in accuracy to items misspelled in stressed and unstressed syllables. The direction of the correlation suggests that the participants with poorer vocabularies were helped most by suprasegmental phonology in detecting misspellings, which is consistent with the finding of greater variance in lexical and spelling decision performance accounted for by segmental phonological feedback in less skilled readers and spellers (Harris and Perfetti, under review).

We had no predictions regarding the relationship of predictability with individual differences, determining it is as likely that sensitivity to context increases as reading skill improves as it is that it decreases. Our correlational analyses showed no association of predictability with individual differences, suggesting that both may be true. Some highly skilled readers may have learned to pay increased attention to unexpected words, and others may find that their skill makes it unnecessary to modify behavior based on predictability. And the opposite may be true, as well: some less skilled readers may use predictability as a cue to help compensate for other deficits, whereas insensitivity to contextual cues may be a driver of poor reading skill for others. Our failure to find a correlation of the predictability effect with individual difference measures is consistent with such a scenario.

Spelling skill was the only individual difference measure that reliably predicted success at detecting omitted words, with two of the three subcomponents of the spelling assessment significantly correlated with omissions accuracy. Spelling ability and omissions detection both require an attention to detail, which explains their correlation. Interestingly, spelling skill was not the most reliable predictor of misspelling detection. This distinction belongs to vocabulary size, which suggests that having complete lexical representations of many words is more helpful in spotting errors while reading in context than is having highly specified orthographic representations for the words in one’s mental lexicon, whatever its size.

## Experiment 2: Reading for Errors

In Experiment 1, participants were asked to both find errors and comprehend content while reading the experimental passage. In Experiments 2 and 3, we manipulate the instructions of the task to test our explanation of the Experiment 1 findings. If we are correct that linguistic context facilitates early activation of lexical stress patterns, thus making stress available to the reader as an error-detection aid, then we should be able to raise or lower error-detection rates by making informational content more or less relevant to successful task completion. In Experiment 2, we emphasize error detection and deemphasize comprehension in the task instructions, with the expectation that the effects of predictability and, critically, lexical stress on error detection rates will be diminished.

### Methods

#### Participants

Participants were 52 Introduction to Psychology students at the University of Pittsburgh who had not participated in Experiment 1. All spoke English at a native or near-native level, and received class credit for their participation.

#### Design and Materials

The experimental design, items, and passages were identical to those in Experiment 1.

#### Procedure

The procedure was identical to that of Experiment 1; only the task instructions the experimenter read aloud to participants (with participants reading along) were altered. Whereas the Experiment 1 instructions informed participants, “You will be asked some simple comprehension questions at the end of the exercise…Please read at a natural pace,” the Experiment 2 instructions read, “Remember, your priority is to detect as many errors as possible, *not* to attain a deep understanding of the material.” No mention was made of post-exercise comprehension questions.

### Results

Task performance measures for Experiment 2 are given in **Table 3**. Five of the 52 participants failed to accurately answer at least three of the four comprehension questions; however, this was not a criterion for removal from analyses as in Experiment 1, because we actively dissuaded participants in Experiment 2 from reading for comprehension. Three of the 80 items (3.75%) were removed from analyses for receiving accuracy rates at or below chance across Experiments 2 and 3. Two of these were misspelled in unstressed syllables and one was misspelled in the stressed syllable. In their response to the feedback question about the purpose of the exercise, 29 of the 52 participants (55.77%) indicated that they were skeptical of the stated purpose, i.e., to find mistakes. Typical answers to the question, “What do you think is the purpose of this experiment?” include, “See how much people absorb when they have a different goal than simple comprehension,” and, “To test a person’s ability to comprehend information while doing something else, so multitasking.”

**Table 3:** Performance Outcomes for Experiment 2.

Measure	Min	Max	Mean	Std. Dev.
Misspellings accuracy	60.00	100.00	83.54	10.55
Repetitions accuracy	0.00	100.00	53.65	24.26
Omissions accuracy	20.00	90.00	57.31	20.97
No. false alarms	0.00	11.00	2.25	2.01
Comprehension questions acc.	25.00	100.00	87.98	18.18

*N = 52. *No. false alarms* refers to the number of times participants identified correctly spelled words as misspelled*.

**FIGURE 2:**
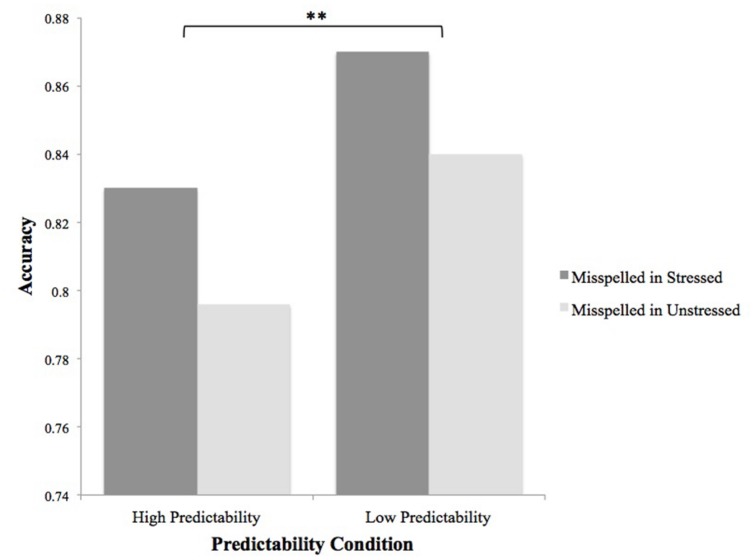
**Main effect of predictability on accuracy in Experiment 2.** Data for subject means is shown; the effect was marginally significant by items. ^∗∗^*p* ≤ 0.01.

Subject- (*F*s) and item- (*F*i) level ANOVAs were performed on spelling error detection accuracy data. There was no main effect of stress status [*F*s(1,51) = 2.82, *p* = 0.1; *F*i(1,153) = 1.64, *p* > 0.1]. A main effect of predictability was significant by subjects [*F*s(1,51) = 9.63, *p* < 0.01, $\eta_{p}^{2}$ = 0.159] and marginal by items [*F*i(1,153) = 3.56, *p* = 0.06, $\eta_{p}^{2}$ = 0.023], with errors more reliably detected in less predictable than in more predictable words. Stress status did not interact with predictability (*F*s and *F*i < 1; **Figure 2**). The results of Experiment 2 are considered further in the Section “General Discussion.”

## Experiment 3: Reading for Information

In Experiment 3, we administer another test of the hypothesis that that linguistic context allows for activation of lexical stress, which can in turn facilitate spelling error detection. This time, we deemphasize error detection and emphasize comprehension in the task instructions, with the expectation that overall error detection will decrease, but errors that are detected will tend to occur in stressed syllables and after low-predictability words.

### Methods

#### Participants

Participants were 63 Introduction to Psychology students at the University of Pittsburgh who had not participated in Experiment 1 or Experiment 2. All spoke English at a native or near-native level, and received class credit for their participation.

#### Design and Materials

The experimental design, items, and passages were identical to those in Experiments 1 and 2.

#### Procedure

The procedure was identical to that of Experiments 1 and 2; again, only the task instructions were altered, this time to downplay the need for error detection. Participants were informed that they were participating in a study on reading comprehension; no mention of proofreading was made, and the practice passage contained no errors. A sentence at the bottom of the instruction sheet stated, “Note: We would appreciate it if you would circle any typos or errors you notice as you read.”

### Results

Task performance measures for Experiment 3 are given in **Table 4**. Error detection was not presented as a requirement for successful task completion in this experiment, and 14 of the 63 participants chose not to circle errors in the passage; their data were not included in analyses. Seven of the remaining 49 participants failed to accurately answer at least three of the four comprehension questions, but their data was included in analyses so that results on this measure could be compared across Experiments 2 and 3. Seventeen of the 80 items had accuracy rates at or below chance, but this alone was not a criterion for removal from analyses, because we did not emphasize error detection in Experiment 3. Rather, only the three items that received chance accuracy in all three experiments were removed from Experiment 3 analyses. In their response to the feedback question about the purpose of the exercise, 29 of the 49 participants (59.18%) indicated that they were skeptical of the stated purpose, i.e., to comprehend the passage. Typical answers to the question, “What do you think is the purpose of this experiment?” include, “To see if one will correct the mistakes found or not while reading for ‘comprehension’,” and, “I think the purpose was actually about how many mistakes we caught in the passage.”

**Table 4:** Performance outcomes for Experiment 3.

Measure	Min	Max	Mean	Std. Dev.
Misspellings accuracy	30.56	95.00	70.99	17.29
Repetitions accuracy	0.00	100.00	31.43	23.45
Omissions accuracy	0.00	80.00	34.49	17.92
No. false alarms	0.00	14.00	1.71	2.30
Comprehension questions acc.	25.00	100.00	88.27	19.83

*N = 49. *No. false alarms* refers to the number of times participants identified correctly spelled words as misspelled*.

**FIGURE 3:**
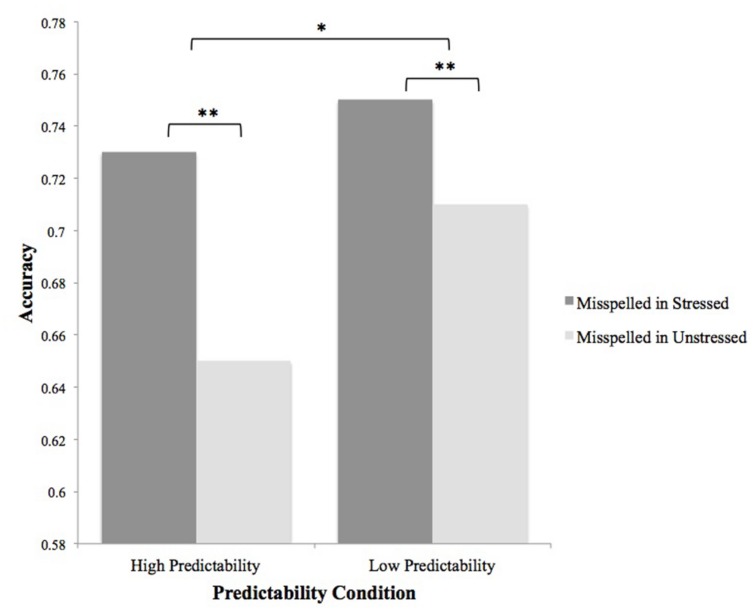
**Main effects of stress status and predictability on accuracy in Experiment 3.** Data for subject means is shown; the predictability effect was not significant by items. ^∗^*p* ≤ 0.05; ^∗∗^*p* ≤ 0.01.

Subject- (*F*s) and item- (*F*i) level ANOVAs were performed on spelling error detection accuracy data. A main effect of stress status was significant by subjects and by items [*F*s(1,48) = 6.79, *p* = 0.01, $\eta_{p}^{2}$ = 0.124; *F*i(1,153) = 5.12, *p* < 0.05, $\eta_{p}^{2}$ = 0.033], with errors more often detected in stressed than in unstressed syllables. A main effect of predictability was significant by subjects [*F*s(1,48) = 5.08, *p* < 0.05, $\eta_{p}^{2}$ = 0.096] but not by items [*F*i(1,153) = 1.49, *p* > 0.1], with errors more often detected in less predictable than in more predictable words. Stress status did not interact with predictability (*F*s and *F*i < 1; **Figure 3**). The results of Experiment 3 are considered further in the Section “General Discussion.”

## General Discussion

**FIGURE 4:**
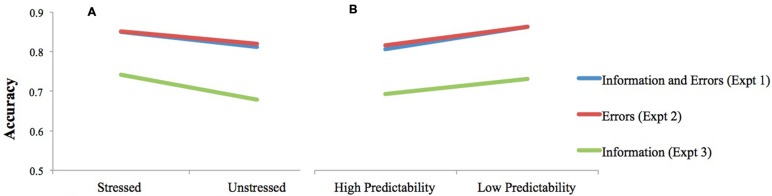
**Misspelling detection accuracy by stress status of misspelled syllable **(A)** and predictability condition **(B)** when task instructions were to read for information and errors (in Experiment 1), errors only (in Experiment 2), or information only (in Experiment 3)**.

Despite participants’ claims when providing feedback on Experiments 2 and 3 to have been skeptical of our stated purpose for the experiment, they seem generally to have taken our instructions at face value during the task itself (**Figure 4**). Error detection rates in Experiment 3, when participants were told to focus on understanding the material, rather than on spotting errors, were about 10 points lower than in Experiments 1 and 2, where error detection was emphasized. Error detection rates between Experiments 1 and 2 were virtually identical, suggesting that the additional task of having to comprehend while reading in Experiment 1 neither hurt nor helped error detection.

**FIGURE 5:**
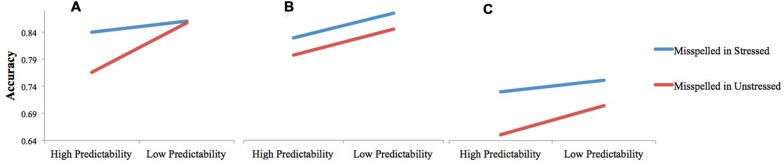
**The relationship between stress status and predictability as it relates to misspelling detection accuracy when participants read the passage **(A)** for both information and errors (Experiment 1); **(B)** for errors only (Experiment 2); or **(C)** for information only (Experiment 3)**.

However, the degree of emphasis put on comprehension in the task instructions *did* influence the role of stress in error detection, and its interaction with predictability (**Figure 5**). Only in Experiment 1, when both understanding the material and detecting errors were emphasized, did stress status and predictability condition interact, such that misspellings were more likely to be detected in a stressed syllable than in an unstressed syllable if the misspelled word was highly predictable from context. This outcome was predicted: we hypothesized that a stress pattern is likelier to be applied to a word in the process of identification during normal reading if a reader is reasonably certain in the milliseconds prior to encountering a word of what it is going to be. A reader’s normally lowered vigilance for predictable words is offset by the attention drawn to the misspelled syllable by stress. In Experiment 3, when error detection was essentially an afterthought, stress status and predictability each independently continued to affect error detection rates, but one no longer moderated the other. It is possible that stress status and linguistic predictability work together efficiently when error detection is a priority, but that the system is not recruited when the stakes of the task are lowered.

The disappearance of stress effects in Experiment 2, when error detection was prioritized, is also in keeping with our hypothesis, because context should not cue stress patterns when context is processed only superficially. Interestingly, context itself did continue to influence error detection in this experiment, such that errors were significantly more likely to be found in unpredictable words. Indeed, the inhibiting property of predictability for finding misspellings is robust in the present research. A number of eye tracking studies have shown that less predictable words tend to receive longer fixations than more predictable words (Ehrlich and Rayner, 1981; Zola, 1984; Schotter et al., 2014), but although longer fixations imply an increased likelihood of noticing a misspelling, eye movements do not provide direct evidence of error detection (Ehrlich and Rayner, 1981, did find that the probability of reporting misspellings following their experiment was higher for misspelled words that had appeared in low-predictability contexts). The role of predictability in error detection has seldom been investigated directly.

A related body of research has asked participants to proofread texts that they were more or less familiar with, under the assumption that every word in a familiar text is more predictable than every word in an unfamiliar text. These studies have led to mixed results, with some reporting increased error detection in more familiar passages (Levy, 1983; Levy and Begin, 1984), others reporting the opposite pattern of results (Pilotti and Chodorow, 2012), and still others reporting an interaction of success at error detection with the method through which familiarity was achieved (Pilotti et al., 2005, 2006). In the experiments reported here, we were able to demonstrate that predictability decreases the likelihood of noticing spelling errors in a text that is being encountered for the first time.

## Conclusion

The present research provides evidence that linguistic predictability modulates the influence of suprasegmental phonology on orthographic processing, which is consistent with our hypothesis that stress can be activated earlier in lexical access when syntax provides cues to upcoming stress patterns. Moreover, stress effects interacted with the predictability of the critical word in context, which adds further support to our hypothesis. If, for example, syntax indicates that the upcoming word will be a noun, the reader will be correct in activating a strong-weak stress pattern approximately 90% of the time (Sereno, 1986; Kelly and Bock, 1988). If context also strongly suggests the upcoming word will be *system*, the reader can activate the strong-weak stress pattern with even greater confidence. Our results show that stress was most beneficial in aiding error detection in highly predictable words, in which errors were less likely to be detected than in less predictable words. That stress did not make a difference for error detection in less predictable words is likely due to the fact that error detection already approached ceiling in those stimuli.

The finding of lexical stress effects in Experiments 1 and 3 is consistent with past studies that found stress is active during silent reading in English (Ashby and Clifton, 2005; Arciuli and Cupples, 2006; Breen and Clifton, 2011), and with studies showing that suprasegmental information can influence our processing of orthography (Drewnowski and Healy, 1982; Goldman and Healy, 1985). The present research cannot, however, answer the question of *how* stress affects orthographic processing. Given the results of Ashby and Clifton (2005), who found that words containing more stressed syllables are fixated longer and more frequently than words containing fewer stressed syllables, a likely explanation for increased error detection in stressed syllables seems tied to the length of fixation times. We showed that predictable words, which are known to receive shorter/fewer fixations, receive less careful orthographic processing than do unpredictable words. Ashby and Clifton suggested that phonological units, including stress units, are assembled for phonological recoding in the completion phase of lexical access. Words with more stressed syllables require more time for the assembly of phonological units, and so are fixated longer before a saccade to the next word is triggered. This explanation accounts for the longer fixations for words with more stressed syllables in their study, but does not account for increased detection of errors in stressed syllables in ours. Indeed, Ashby and Clifton found that fixations of syllables containing stress did not differ from those of unstressed syllables. Further research is necessary to explain why written syllables with stress attached to them are more visually salient than unstressed syllables.

Finally, we found evidence that individual differences in reading-related skills can predict the size of lexical stress effects on orthographic processing during reading in context. Specifically, vocabulary knowledge correlated with the stress effect in Experiment 1, such that participants with poorer vocabularies were helped most by suprasegmental phonology in detecting misspellings. Past research has shown that less skilled readers rely more heavily on segmental phonology during orthographic processing than do more skilled readers, but this is, to our knowledge, the first demonstration that they are also more reliant on suprasegmental information.

## Author Contributions

LH designed this series of experiments and was primarily responsible for the acquisition, analysis, and interpretation of data. Experiment 1 of this research comprises a portion of LH’s doctoral dissertation. CP contributed to the conception and design of the study, and critically revised manuscripts for important intellectual content.

## Conflict of Interest Statement

The authors declare that the research was conducted in the absence of any commercial or financial relationships that could be construed as a potential conflict of interest.
