# Supplementary material for: Lexical Stress and Linguistic Predictability Influence Proofreading Behavior
Source: Front Psychol. 2016 Feb 9;7:96. doi: 10.3389/fpsyg.2016.00096 (PMC4746312; doi:10.3389/fpsyg.2016.00096)
Supplement: Supplementary file 1 [file Image_1.PDF]

APPENDIX A

Experimental stimuli.

Target	Misspelled in Stressed Syllable	Misspelled in Unstressed Syllable
agreement	agreament	agreemint
announcer	annauncer	announcir
beautiful	beoutiful	beautaful
betray	betrey	butray
bleachers	bleechers	bleachurs
business	bisiness	businiss
certainly	cyrtainly	certaenly
colored	culored	colured
colorful	culorful	colorfol
comfort	cumfort	comfert
company	cumpany	compeny
consensus	consynsus	cunsensus
container	contayner	cuntainer
council	coencil	councel
covering	cuvering	coveryng
determine	deturmine	ditermine
discover	discuver	dyscover
divergent	divirgent	dyvergent
diversion	divirsion	dyversion
dynamite	dinamite	dynamyte
easily	eesily	easely
encourage	encoarage	encourege
freedom	freadom	freedum
governor	guvernor	govurner
internal	inturnal	internel
language	lenguage	languege
lovingly	luvingly	lovyngly
machine	machene	mechine
movement	muvement	movemint
personal	pursonal	personel
physical	phisical	physycal
prettiest	prittiest	prettyest
purpose	perpose	purpuse
retreat	retreet	ritreat
reveal	reveel	riveal
service	sirvice	servyce
surgery	sergery	surgury
system	sistem	systim
wonderful	wunderful	wonderfol
worthless	werthless	worthliss
