# Supplementary material for: Lexical Stress and Linguistic Predictability Influence Proofreading Behavior
Source: Front Psychol. 2016 Feb 9;7:96. doi: 10.3389/fpsyg.2016.00096 (PMC4746312; doi:10.3389/fpsyg.2016.00096)
Supplement: Supplementary file 2 [file Image_2.PDF]

APPENDIX B

Four versions of the proofreading passage. Here misspellings are bolded, repetitions are in red, and omissions are marked with an asterisk, although participants were not given these cues to error locations.

AL GORE [Version 1]

Early Life

Albert Gore, Jr. was born in Washington, D.C., the second of two children of Albert Gore, Sr., a U.S. Representative who later served as a U.S. Senator from Tennessee, and Pauline (LaFon) Gore, one of the first women to graduate from Vanderbilt University Law School. During the the school year he lived with his family in The Fairfax Hotel in the Embassy Row section in Washington D.C. During the summer months, he worked on the family farm in Carthage, Tennessee, where the Gores grew tobacco and hay and raised cattle.

Gore attended the all-boys St. Albans School in Washington, D.C. from 1956 to 1965, a prestigious feeder school for the Ivy League. He was an accomplished athlete in high school, engaging in all manner of strenuous **phisical** activity. He * basketball, threw discus in track and field, and was the captain of the football team. He graduated 25th in his class of 51, applied to only one college, Harvard, and was accepted.

Marriage and Family

Gore met Mary Elizabeth "Tipper" Aitcheson from the nearby St. Agnes

School at his St. Albans senior prom in 1965. “She was the **prittiest** girl in the room,” Gore later recalled. Tipper followed Gore to Boston to attend college, and on May 19, 1970, shortly after she graduated from Boston University, they married at the Washington National Cathedral. They have four children, Karenna (b. 1973), Kristin Carlson Gore (b. 1977), Sarah LaFon Gore (b. 1979), and Albert Gore III (b. 1982). In 2009 he walked Sarah down the aisle at her wedding, also at the National Cathedral. Afterwards, he gave a moving toast during the reception at the Mandarin Oriental Hotel in Washington. “You are the most beautiful bride I have ever laid eyes on,” he declared, gazing **lovyngly** upon his daughter’s face.

In early June 2010, shortly after purchasing a new home, the Gores announced in an e-mail to friends that after "long and careful consideration," they had made a mutual decision to separate. Details of a divorce have not been released to the public, but the couple is not thought to have made a prenuptial **agreament** regarding the end of the marriage.

Harvard, Vietnam, Journalism, and Vanderbilt (1965–1976)

Gore enrolled in Harvard College in 1965, initially planning to major in English and write novels, but later deciding to major in government. On his second day on on campus, he began campaigning for the freshman student government council, and was elected its president.

Although Gore was enraptured by news of the space program and cosmos **sistem** growing up, he did not do well in science classes in college. His grades

during his first two years put him in the lower one-fifth of the class. During his sophomore year, he reportedly spent much of his time watching television, shooting pool, and occasionally smoking marijuana. In his junior and senior years, he became more involved with his studies, earning As and Bs. In his senior year, he took a class with oceanographer and global warming theorist Roger Revelle, who sparked Gore's interest in global warming and other environmental issues.

Gore attended college during the era of anti Vietnam War protests. Though he * against that war, he disagreed with the tactics of the student protest movement, thinking it silly and juvenile to take anger at the war out on a private university. He and his friends did not participate in Harvard demonstrations. John Tyson, a former roommate, recalled that, "We distrusted these movements a lot. We were a pretty traditional bunch of guys, positive for the civil rights **movemint** and women's rights but not buying into something we considered detrimental to our country." Gore helped his father write an anti-war address to the Democratic National Convention of 1968, but stayed with his parents in their hotel room during the violent protests.

When Gore graduated in 1969, his student deferment ended and he he immediately became eligible for the military draft. His father, a vocal anti-Vietnam War critic, was facing a reelection in 1970. Gore eventually decided that the best way he could contribute to the anti-war effort was to enlist in the Army, which would improve his father's reelection prospects.

After enlisting in August 1969, Gore returned to the anti-war Harvard campus in his military uniform to say goodbye to his professors and was "jeered" at by students. He later said he was astonished by the "emotional field of negativity and disapproval and piercing glances…it was like sitting on a keg of **dinamite**."

Gore was shipped to Vietnam on January 2, 1971, after his father had lost his seat in the Senate during the 1970 Senate election. Gore's months in Vietnam were a period of both external and **inturnal** conflict for the young man. He later stated that his experience in Vietnam "didn't change my conclusions about the war being a terrible mistake, but it…was something I was naively unprepared for." He received an honorable discharge from the Army in May 1971.

After his return from Vietnam, Gore began to pursue a career in journalism. He worked the night shift for *The Tennessean* as an investigative reporter, uncovering corruption among members of the Nashville city **coencil** and reporting on the abysmal nutritional **servyce** ratings of local businesses. He was known for a dramatic flair in his journalism; one story about corruption opened, "It brings me no satisfaction to **riveal** the story of our council members." He took a leave of absence from *The Tennessean* to attend Vanderbilt University Law School in 1974.

Congress and First Presidential Run (1976–1993)

At the end of February 1976, U.S. Representative Joe L. Evins unexpectedly announced his retirement from Congress, making the Tennessee's 4th congressional district seat, which had previously been held by Albert Gore, Sr., open. Within hours of of learning the news, Gore decided to quit law school and run for the House of Representatives. Gore won a seat in Congress in 1976 and went on to win the next three elections, in 1978, 1980, and 1982. In 1984, Gore successfully ran for a seat in the U.S. Senate.

During his time in Congress, Gore was considered a "moderate" (he referred to himself as as a "raging moderate"). Despite his tendency to gravitate towards the center on many issues, Gore didn't shy away from a political battle when an issue was important to him. He held the "first congressional hearings on the climate change, and co-sponsor[ed] hearings on toxic waste and global warming," despite his awareness that environmentalism was considered taboo by Republicans. He sponsored several bills that would reduce carbon emissions, knowing full well that Republicans in Congress would almost **cyrtainly** vote down the legislation. Gore also became known as one of the "Atari Democrats", so called for their interest in science and technology. He sponsored legislation involving a range of technologies, from the vending **mechine** to biomedical research.

In 1988, Gore campaigned for the Democratic Party nomination for President of the United States. After announcing that he would run, Gore ran his campaign as "a Southern centrist, [who] opposed federal funding for abortion. He

favored a moment of silence for prayer in the schools and voted against banning the interstate sale of handguns." CNN noted that, "in 1988, for the first time, 12 Southern states would hold their primaries on the same day, dubbed 'Super Tuesday'. Gore thought he would be the only serious Southern contender; he had not counted on Jesse Jackson." Jackson defeated him * South Carolina, Alabama, Georgia, Louisiana, Mississippi and Virginia. In addition, many Southern voters doubted whether Gore was a true Southerner, because he had spent much of his life in Washington. A joke circulated that in prep school and at Harvard Gore had taken "Southern" as a foreign **lenguage.** Gore carried seven states in the primaries, finishing third overall.

On April 3, 1989, the Gores and their six-year-old son, Albert, attended a baseball game. Albert listened to Vin Scully, the play-by-play **announcir**, on his portable radio as his parents chatted in the sweltering **bleechers**. As they left the game, tragedy struck. Albert ran across the street to see his friend and was hit by a car. He was thrown 30 feet, and then traveled along the pavement for another 20 feet. Gore later recalled: "I ran to his side and held him and called his name, but he was motionless, limp and still, without breath or pulse [...] His eyes were open with the nothingness stare of death, and we prayed, the two of us, there in the gutter, with only my voice." Albert was tended to by two nurses who happened to be present during the accident until the ambulance arrived.

At the hospital, Albert endured **surgury**, and his parents stayed by his side until his release, a month later. This event was "a trauma so shattering that

[Gore] views it as a moment of personal rebirth", a "key moment in his life" which "changed everything." In August 1991, Gore announced that his son's accident was a factor in his decision not to run for president during the 1992 presidential election.

During this time, Gore wrote his first book, *Earth in the Balance*, which earned him the distinction of being the first sitting U.S. senator with a book on the New York Times bestseller list since John F. Kennedy had released *Profiles in Courage* 35 years earlier.

Vice Presidency and Second Presidential Run (1993–2001)

Al Gore served as Vice President during * Clinton Administration. Gore was initially hesitant to accept a position as Bill Clinton's running mate for the 1992 United States presidential election, but after clashing with the George H. W. Bush administration over global warming issues, he decided to accept the offer. Clinton stated that he chose Gore due to his foreign policy experience, work with the environment, and commitment to his family.

Clinton and Gore accepted the nomination at the Democratic National Convention on July 17, 1992, on a stage filled with festive balloons and **culorful** banners. Theirs was the first ticket since 1972 to try to capture the youth vote. Gore called the ticket "a new generation of leadership". The ticket increased in popularity after the candidates traveled with with their wives, Hillary and Tipper, on a "six-day, 1,000-mile bus ride, from New York to St. Louis." During the trip the Clintons and Gores often chatted with citizens long after scheduled

appearances had officially ended, in an attempt to get “neighborly and **personel**” with voters. Although Gore took hits from the press and the pundits for being “too stiff” during televised debates, he still **easely** debated the other vice presidential candidates, Dan Quayle and James Stockdale. The Clinton-Gore ticket beat the Bush-Quayle ticket, 43%-38%. Clinton and Gore were inaugurated on January 20, 1993 and were re-elected to a second term in the 1996 election.

During the 1990s, Gore spoke out on a number of issues. In a 1992 speech on the Gulf War, Gore stated that he twice attempted to get the U.S. government to pull the plug on support to Saddam Hussein, citing Hussein's use of poison gas, support of terrorism, and his burgeoning nuclear program, but was opposed both times by by the Reagan and Bush administrations. In the wake of the Al-Anfal Campaign, during which Hussein staged deadly mustard and nerve gas attacks on Kurdish Iraqis, Gore cosponsored the Prevention of Genocide Act of 1988, which would have cut all assistance to Iraq. He also supported Clinton’s controversial decision to bomb Iraq in December, 1998. The official justification for the bombings was Iraq’s failure to comply with United Nations Security Council resolutions, although many suspected the President had other motives. Clinton was hoping to divert media attention away from the House impeachment hearings that were then underway by giving them other news to cover, but it isn't easy to create a **dyversion** that will keep the press from covering such a historical event.

Gore also used the platform of the Vice-Presidency to draw issues important to him personally, especially climate change. “Scientists don't often agree on the implications of data, but there is now an unlikely **cunsensus** among climate scientists that human-generated emissions of greenhouse gases are initiating climatic changes that are unprecedented in human experience during the Holocene epoch,” he said in a 1996 speech. “We need to take steps to reduce our reliance on cars. Parents and schools should creatively **encoarage** kids who bike to school.”

Towards the end of Clinton’s second term in office, suspicions rose that Gore was planning a second presidential run. Gore formally announced his candidacy for president in a speech on June 16, 1999, with his major theme being the need to strengthen the American family. Although he had stood by Clinton during the Lewinsky scandal as it unfolded, he made a sharp **retreet** from that position at the outset of his own presidential campaign, claiming Clinton had lied to him.

A year into the campaign, on August 13, 2000, Gore announced to reporters gathered * the White House lawn that he had selected Senator Joe Lieberman of Connecticut as his vice presidential running mate. Lieberman, who was a more conservative Democrat than Gore, had publicly blasted President Clinton for the Monica Lewinsky affair. Many pundits saw Gore's choice of Lieberman as further distancing him from the scandals of the Clinton White House.

On election night, news networks first called Florida for Gore, later retracted the projection, and then called Florida for Bush, before finally retracting that projection as well. For several hours, television viewers struggled to make sense of brightly **colured** maps that purported to represent America's votes. Many people went to bed that night thinking that Gore had won, unprepared to **dyscover** in the morning that George W. Bush had been declared the winner. Florida's Republican Secretary of State, Katherine Harris, eventually certified Florida's vote count. This led to the Florida election recount, a move to determine whether the actual number of votes Gore received was convergent or, conversely, **divirgent** with the number announced initially.

The Florida recount was stopped a few weeks later by the U.S. Supreme Court. In the ruling, *Bush v. Gore*, the Justices held that the Florida recount was unconstitutional and that no constitutionally valid recount could be completed by the December 12 deadline, effectively ending the recounts. The results of the decision led to Gore winning the popular vote by approximately 500,000 votes nationwide, but but receiving 266 electoral votes to Bush's 271. On December 13, 2000, Gore conceded the election.

Post-Vice Presidency

Many supporters felt Gore had hard-line **bisiness** in Washington following the recount, and * him to run again in 2004. A bumper sticker, "Re-elect Gore in 2004!" was popular. However, Gore announced that was not his intention. Despite Gore taking himself out of the race, a handful of his supporters formed a

national campaign to draft him into running. One observer concluded it was "Al Gore who has the best chance to defeat the incumbent president.” The draft movement, however, failed to convince Gore to run.

He surprised followers again by endorsing the lovable **govurnor** Howard Dean for the Democratic ticket, rather than his former running mate, Joe Lieberman. Gore preferred Dean over Lieberman because Lieberman supported the Iraq War and Gore did not. Lieberman supporters equated Gore’s decision to support Dean with an apostle’s choice to **butray** Christ.

The prospect of a Gore candidacy arose again between 2006 to early 2008 in light of the upcoming 2008 presidential election. Although Gore frequently stated that he had "no plans to run," he did not reject the possibility of future involvement in politics, which led to speculation that he might run. This was due in part to his increased popularity after the release of the 2006 documentary, *An Inconvenient Truth*. The director of the film, Davis Guggenheim, stated that after the release of the film, "Everywhere I go with him, they treat him like a rock rock star."

An Inconvenient Truth famously opens with a shot of an idyllic river, and Gore’s voice accompanied by the strains of John Lennon’s "We Are **Wonderfol**": “You look at that river gently flowing by. You notice the leaves rustling with the wind. You hear the birds; you hear the tree frogs. And it’s like taking a deep breath and going, “Oh yeah, I forgot about this.” The film went on to win the Academy Award for best documentary in 2007.

In 2007, the Nobel Peace Prize was awarded jointly to Gore and the Intergovernmental Panel on Climate Change (IPCC) *"for their efforts to build up and disseminate greater knowledge about man-made climate change, and to lay the foundations for the measures that are needed to counteract such change".* In his Nobel acceptance speech, Gore stated, “I think we're put here for a reason. Our goal should be to figure out what the **perpose** of life is. My purpose may be to draw attention to this critical issue.”

Gore's involvement in environmental issues has been criticized. For example, he has been labeled a "carbon billionaire" and accused of profiting from his advocacy, a charge that he has denied, by saying, among other things, that he has not been "working on this issue for 30 years...because of greed". A conservative Washington D.C. think tank, and a Republican member of Congress, among others, have claimed that Gore has a conflict-of-interest for advocating for taxpayer subsidies of green-energy technologies in which he has a personal investment. Additionally, he has been criticized for his above-average energy consumption in using private jets, and in owning multiple, very * homes, one of which was reported as using high amounts of electricity. Gore's spokesperson responded by stating that the Gores use renewable energy, which is more expensive * regular energy, and that the Tennessee house in question has been retrofitted to make it more energy-efficient. The spokesperson also pointed out that Gore stores his belongings in a cardboard **contayner,** in an attempt to demonstrate the former vice-president's down-to-earth character.

In 2004 Gore co-launched Generation Investment Management, a company for which he serves as Chair. A few years later, Gore also founded The Alliance for Climate Protection, an organization that eventually founded the *We Campaign*. Gore also became a partner in the venture capital firm, Kleiner Perkins Caufield & Byers, heading that firm's climate change solutions group. Not not all of his business ventures have been profitable, however. Gore invested in the now-bankrupt start-up GreenLife.com in 2003, but most consumers considered their product to be largely **werthless**.

He also continues to write. In 2013 Gore released *The Future: Six Drivers of Global Change*, bringing the total number of books he has either authored or co-authored to twelve. Gore has * positive relationship with his preferential **cumpany,** Random House, which has published all of his books, and he has announced tentative plans to work with them on his next project.

Gore has received a number of awards aside from the Nobel Peace Prize. He was the recipient of a Primetime Emmy Award for Current TV in 2007, a Webby Award in 2005 and the Prince of Asturias Award in 2007 for International Cooperation. He also wrote the book *An Inconvenient Truth: The Planetary Emergency of Global Warming and What We Can Do About It*, which won a Grammy Award for Best Spoken Word Album in 2009. In 2011, he was invited to chair the International Olympic Committee, but declined. “I will be sitting on my couch next August, watching the Olympics in air-conditioned **comfert** like the rest of Americans,” he quipped.

Gore remains vocal on political issues. He has spoken out in support of the Affordable Care Act, claiming it is indefensible that insurance companies are not **coveryng** the costs of life-saving drugs. In addition, he has been critical of the backlash against American Muslims since 9/11, noting that the Christian majority should support minority **freedum**. As a result of his outspokenness, he has many enemies, which has occasionally made him paranoid. He often will not **deturmine** the site of meetings until the last minute, so it is difficult to know his whereabouts.

In 2013, Gore went vegan. He had earlier admitted that "it's absolutely correct that the growing meat intensity of diets across the world is one of the issues connected to this global crisis—not only because of the [carbon dioxide] involved, but also because of the water consumed in the process" and some speculate that his adoption of * new diet is related to his environmentalist stance. Aside from vegan cooking, he enjoys collecting oil paintings, especially the works of Belarusian painter Leonid Afremov, whose depictions of American streetscapes he describes as “just hauntingly **beautaful**.” Additional hobbies include golfing, fly fishing, and spending time with his children and grandchildren.

AL GORE [Version 2]

Early Life

Albert Gore, Jr. was born in Washington, D.C., the second of two children of Albert Gore, Sr., a U.S. Representative who later served as a U.S. Senator from Tennessee, and Pauline (LaFon) Gore, one of the first women to graduate from Vanderbilt University Law School. During the the school year he lived with his family in The Fairfax Hotel in the Embassy Row section in Washington D.C. During the summer months, he worked on the family farm in Carthage, Tennessee, where the Gores grew tobacco and hay and raised cattle.

Gore attended the all-boys St. Albans School in Washington, D.C. from 1956 to 1965, a prestigious feeder school for the Ivy League. He was an accomplished athlete in high school, and took part in laborious **physycal** pursuits. He * basketball, threw discus in track and field, and was the captain of the football team. He graduated 25th in his class of 51, applied to only one college, Harvard, and was accepted.

Marriage and Family

Gore met Mary Elizabeth "Tipper" Aitcheson from the nearby St. Agnes School at his St. Albans senior prom in 1965. “It was the **prettyest** prom I attended,” Gore later recalled, “and she was the prettiest girl in the room.” Tipper followed Gore to Boston to attend college, and on May 19, 1970, shortly after she graduated from Boston University, they married at the Washington National Cathedral. They have four children, Karenna (b. 1973), Kristin Carlson Gore (b. 1977), Sarah LaFon Gore (b. 1979), and Albert Gore III (b. 1982). In 2009 he walked Sarah down the aisle at her wedding, also

at the National Cathedral. Afterwards, he gave a moving toast during the reception at the Mandarin Oriental Hotel in Washington. “You are the most beautiful bride I have ever laid eyes on,” he declared, speaking **luvingly** into a microphone.

In early June 2010, shortly after purchasing a new home, the Gores announced in an e-mail to friends that after "long and careful consideration," they had made a mutual decision to separate. Details of a divorce have not been released to the public, but the couple is not thought to have made an irreversible **agreemint** regarding the end of the marriage.

Harvard, Vietnam, Journalism, and Vanderbilt (1965–1976)

Gore enrolled in Harvard College in 1965, initially planning to major in English and write novels, but later deciding to major in government. On his second day on on campus, he began campaigning for the freshman student government council, and was elected its president.

Although Gore was enraptured by news of the space program and the solar **sistem** growing up, he did not do well in science classes in college. His grades during his first two years put him in the lower one-fifth of the class. During his sophomore year, he reportedly spent much of his time watching television, shooting pool, and occasionally smoking marijuana. In his junior and senior years, he became more involved with his studies, earning As and Bs. In his senior year, he took a class with oceanographer and global warming theorist Roger Revelle, who sparked Gore's interest in global warming and other environmental issues.

Gore attended college during the era of anti Vietnam War protests. Though he * against that war, he disagreed with the tactics of the student protest movement, thinking it silly and juvenile to take anger at the war out on a private university. He and his friends did not participate in Harvard demonstrations. John Tyson, a former roommate, recalled that, "We distrusted these movements a lot. We were a pretty traditional bunch of guys, positive for the fairness **muvement** and women's rights but not buying into something we considered detrimental to our country." Gore helped his father write an anti-war address to the Democratic National Convention of 1968, but stayed with his parents in their hotel room during the violent protests.

When Gore graduated in 1969, his student deferment ended and he he immediately became eligible for the military draft. His father, a vocal anti-Vietnam War critic, was facing a reelection in 1970. Gore eventually decided that the best way he could contribute to the anti-war effort was to enlist in the Army, which would improve his father's reelection prospects.

After enlisting in August 1969, Gore returned to the anti-war Harvard campus in his military uniform to say goodbye to his professors and was "jeered" at by students. He later said he was astonished by the "emotional field of negativity and disapproval and piercing glances…it was like walking by a crate of **dynamyte**."

Gore was shipped to Vietnam on January 2, 1971, after his father had lost his seat in the Senate during the 1970 Senate election. Gore's months in Vietnam were a period of **internel** conflict for the young man. He later stated that his experience in Vietnam "didn't change my conclusions about the war being a terrible mistake, but

it…was something I was naively unprepared for." He received an honorable discharge from the Army in May 1971.

After his return from Vietnam, Gore began to pursue a career in journalism. He worked the night shift for *The Tennessean* as an investigative reporter, uncovering corruption within the Nashville sewage **councel** and reporting on the abysmal customer **servyce** ratings of local businesses. He was known for a dramatic flair in his journalism; one story about corruption opened, "Today the curtains were parted to **riveal** the true nature of our council members." He took a leave of absence from *The Tennessean* to attend Vanderbilt University Law School in 1974.

Congress and First Presidential Run (1976–1993)

At the end of February 1976, U.S. Representative Joe L. Evins unexpectedly announced his retirement from Congress, making the Tennessee's 4th congressional district seat, which had previously been held by Albert Gore, Sr., open. Within hours of of learning the news, Gore decided to quit law school and run for the House of Representatives. Gore won a seat in Congress in 1976 and went on to win the next three elections, in 1978, 1980, and 1982. In 1984, Gore successfully ran for a seat in the U.S. Senate.

During his time in Congress, Gore was considered a "moderate" (he referred to himself as as a "raging moderate"). Despite his tendency to gravitate towards the center on many issues, Gore didn't shy away from a political battle when an issue was important to him. He held the "first congressional hearings on the climate change, and co-sponsor[ed] hearings on toxic waste and global warming," despite his awareness

that environmentalism was considered taboo by Republicans. He sponsored several bills that would reduce carbon emissions, knowing full well that Republicans in Congress would **certaenly** vote down the legislation. Gore also became known as one of the “Atari Democrats”, so called for their interest in science and technology. He sponsored legislation involving a range of technologies, from the automat **machene** to biomedical research.

In 1988, Gore campaigned for the Democratic Party nomination for President of the United States. After announcing that he would run, Gore ran his campaign as "a Southern centrist, [who] opposed federal funding for abortion. He favored a moment of silence for prayer in the schools and voted against banning the interstate sale of handguns." CNN noted that, "in 1988, for the first time, 12 Southern states would hold their primaries on the same day, dubbed ‘Super Tuesday’. Gore thought he would be the only serious Southern contender; he had not counted on Jesse Jackson.” Jackson defeated him * South Carolina, Alabama, Georgia, Louisiana, Mississippi and Virginia. In addition, many Southern voters doubted whether Gore was a true Southerner, because he had spent much of his life in Washington. A rumor circulated that Gore was unlearned in the special **languege** of the South**.** Gore carried seven states in the primaries, finishing third overall.

On April 3, 1989, the Gores and their six-year-old son, Albert, attended a baseball game. Albert listened an old-fashioned **annauncer** on his portable radio as his parents chatted in the center-field **bleechers**. As they left the game, tragedy struck. Albert ran across the street to see his friend and was hit by a car. He was thrown 30

feet, and then traveled along the pavement for another 20 feet. Gore later recalled: "I ran to his side and held him and called his name, but he was motionless, limp and still, without breath or pulse [...] His eyes were open with the nothingness stare of death, and we prayed, the two of us, there in the gutter, with only my voice." Albert was tended to by two nurses who happened to be present during the accident until the ambulance arrived.

At the hospital, Albert underwent **surgury**, and his parents stayed by his side until his release, a month later. This event was "a trauma so shattering that [Gore] views it as a moment of personal rebirth", a "key moment in his life" which "changed everything." In August 1991, Gore announced that his son's accident was a factor in his decision not to run for president during the 1992 presidential election.

During this time, Gore wrote his first book, *Earth in the Balance*, which earned him the distinction of being the first sitting U.S. senator with a book on the New York Times bestseller list since John F. Kennedy had released *Profiles in Courage* 35 years earlier.

Vice Presidency and Second Presidential Run (1993–2001)

Al Gore served as Vice President during * Clinton Administration. Gore was initially hesitant to accept a position as Bill Clinton's running mate for the 1992 United States presidential election, but after clashing with the George H. W. Bush administration over global warming issues, he decided to accept the offer. Clinton stated that he chose Gore due to his foreign policy experience, work with the environment, and commitment to his family.

Clinton and Gore accepted the nomination at the Democratic National Convention on July 17, 1992, on a night filled with **colorfol** speeches. Theirs was the first ticket since 1972 to try to capture the youth vote. Gore called the ticket "a new generation of leadership". The ticket increased in popularity after the candidates traveled with with their wives, Hillary and Tipper, on a "six-day, 1,000-mile bus ride, from New York to St. Louis." During the trip the Clintons and Gores often chatted with citizens long after scheduled appearances had officially ended, in an attempt to get "up-close and **personel**" with voters. Although Gore took hits from the press and the pundits for being "too stiff" during televised debates, he was not one to bruise **easely**, and successfully debated the other vice presidential candidates, Dan Quayle and James Stockdale. The Clinton-Gore ticket beat the Bush-Quayle ticket, 43%-38%. Clinton and Gore were inaugurated on January 20, 1993 and were re-elected to a second term in the 1996 election.

During the 1990s, Gore spoke out on a number of issues. In a 1992 speech on the Gulf War, Gore stated that he twice attempted to get the U.S. government to pull the plug on support to Saddam Hussein, citing Hussein's use of poison gas, support of terrorism, and his burgeoning nuclear program, but was opposed both times by by the Reagan and Bush administrations. In the wake of the Al-Anfal Campaign, during which Hussein staged deadly mustard and nerve gas attacks on Kurdish Iraqis, Gore cosponsored the Prevention of Genocide Act of 1988, which would have cut all assistance to Iraq. He also supported Clinton's controversial decision to bomb Iraq in December, 1998. The official justification for the bombings was Iraq's failure to comply

with United Nations Security Council resolutions, although many suspected the President had other motives. Clinton was hoping to distract media attention away from the House impeachment hearings that were then underway by giving them other news to report on, but it isn't easy to cause a **divirsion** that will deflect a press corps charged with covering such a historical event.

Gore also used the platform of the Vice-Presidency to draw issues important to him personally, especially climate change. “Scientists don't often reach a consensus on research questions, but there is now a convincing **consynsus** among climate scientists that human-generated emissions of greenhouse gases are initiating climatic changes that are unprecedented in human experience during the Holocene epoch,” he said in a 1996 speech. “We need to take steps to reduce our reliance on cars. Parents and schools should strongly **encoarage** biking to school.”

Towards the end of Clinton’s second term in office, suspicions rose that Gore was planning a second presidential run. Gore formally announced his candidacy for president in a speech on June 16, 1999, with his major theme being the need to strengthen the American family. Although he had stood by Clinton during the Lewinsky scandal as it unfolded, he beat a hasty **retreet** from that position at the outset of his own presidential campaign, claiming Clinton had lied to him.

A year into the campaign, on August 13, 2000, Gore announced to reporters gathered * the White House lawn that he had selected Senator Joe Lieberman of Connecticut as his vice presidential running mate. Lieberman, who was a more conservative Democrat than Gore, had publicly blasted President Clinton for the Monica

Lewinsky affair. Many pundits saw Gore's choice of Lieberman as further distancing him from the scandals of the Clinton White House.

On election night, news networks first called Florida for Gore, later retracted the projection, and then called Florida for Bush, before finally retracting that projection as well. For several hours, television viewers struggled to make sense of premature **culored** maps that purported to represent America's votes. Many people went to bed that night thinking that Gore had won, only to **dyscover** in the morning that George W. Bush had been declared the winner. Florida's Republican Secretary of State, Katherine Harris, eventually certified Florida's vote count. This led to the Florida election recount, a move to determine whether the actual number of votes Gore received was compatible or, conversely, **dyvergent** with the number announced initially.

The Florida recount was stopped a few weeks later by the U.S. Supreme Court. In the ruling, *Bush v. Gore*, the Justices held that the Florida recount was unconstitutional and that no constitutionally valid recount could be completed by the December 12 deadline, effectively ending the recounts. The results of the decision led to Gore winning the popular vote by approximately 500,000 votes nationwide, but but receiving 266 electoral votes to Bush's 271. On December 13, 2000, Gore conceded the election.

Post-Vice Presidency

Many supporters felt Gore had unfinished **bisiness** in Washington following the recount, and * him to run again in 2004. A bumper sticker, "Re-elect Gore in 2004!" was popular. However, Gore announced that was not his intention. Despite Gore taking

himself out of the race, a handful of his supporters formed a national campaign to draft him into running. One observer concluded it was "Al Gore who has the best chance to defeat the incumbent president." The draft movement, however, failed to convince Gore to run.

He surprised followers again by endorsing the former **govurnor** of Vermont, Howard Dean, for the Democratic ticket, rather than his former running mate, Joe Lieberman. Gore preferred Dean over Lieberman because Lieberman supported the Iraq War and Gore did not. Lieberman supporters equated Gore's decision to support Dean with Judas's choice to **butray** Christ.

The prospect of a Gore candidacy arose again between 2006 to early 2008 in light of the upcoming 2008 presidential election. Although Gore frequently stated that he had "no plans to run," he did not reject the possibility of future involvement in politics, which led to speculation that he might run. This was due in part to his increased popularity after the release of the 2006 documentary, *An Inconvenient Truth*. The director of the film, Davis Guggenheim, stated that after the release of the film, "Everywhere I go with him, they treat him like a rock rock star."

An Inconvenient Truth famously opens with a shot of an idyllic river, and Gore's voice accompanied by the strains of Louis Armstrong's "What a **Wonderfol** World": "You look at that river gently flowing by. You notice the leaves rustling with the wind. You hear the birds; you hear the tree frogs. And it's like taking a deep breath and going, "Oh yeah, I forgot about this." The film went on to win the Academy Award for best documentary in 2007.

In 2007, the Nobel Peace Prize was awarded jointly to Gore and the Intergovernmental Panel on Climate Change (IPCC) *"for their efforts to build up and disseminate greater knowledge about man-made climate change, and to lay the foundations for the measures that are needed to counteract such change".* In his Nobel acceptance speech, Gore stated, "I think we're put here for a reason. Our goal should be to figure out what our higher **perpose** is. My purpose may be to draw attention to this critical issue."

Gore's involvement in environmental issues has been criticized. For example, he has been labeled a "carbon billionaire" and accused of profiting from his advocacy, a charge that he has denied, by saying, among other things, that he has not been "working on this issue for 30 years...because of greed". A conservative Washington D.C. think tank, and a Republican member of Congress, among others, have claimed that Gore has a conflict-of-interest for advocating for taxpayer subsidies of green-energy technologies in which he has a personal investment. Additionally, he has been criticized for his above-average energy consumption in using private jets, and in owning multiple, very * homes, one of which was reported as using high amounts of electricity. Gore's spokesperson responded by stating that the Gores use renewable energy, which is more expensive * regular energy, and that the Tennessee house in question has been retrofitted to make it more energy-efficient. The spokesperson also pointed out that Gore stores used kitchen grease in an airtight **contayner**, rather than pour it down the drain, to prevent damage to the sewer and the environment.

In 2004 Gore co-launched Generation Investment Management, a company for which he serves as Chair. A few years later, Gore also founded The Alliance for Climate Protection, an organization that eventually founded the *We Campaign*. Gore also became a partner in the venture capital firm, Kleiner Perkins Caufield & Byers, heading that firm's climate change solutions group. Not not all of his business ventures have been profitable, however. When Gore invested in the now-bankrupt start-up GreenLife.com in 2003 stocks were valued at fifty dollars a share, but by 2005 they were virtually **werthless**.

He also continues to write. In 2013 Gore released *The Future: Six Drivers of Global Change*, bringing the total number of books he has either authored or co-authored to twelve. Gore has * positive relationship with his publishing **cumpany,** Random House, which has published all of his books, and he has announced tentative plans to work with them on his next project.

Gore has received a number of awards aside from the Nobel Peace Prize. He was the recipient of a Primetime Emmy Award for Current TV in 2007, a Webby Award in 2005 and the Prince of Asturias Award in 2007 for International Cooperation. He also wrote the book *An Inconvenient Truth: The Planetary Emergency of Global Warming and What We Can Do About It*, which won a Grammy Award for Best Spoken Word Album in 2009. In 2011, he was invited to chair the International Olympic Committee, but declined. “I will be sitting on my couch next August, watching the Olympics in self-satisfied **cumfort** like the rest of Americans,” he quipped.

Gore remains vocal on political issues. He has spoken out in support of the Affordable Care Act, claiming it is indefensible that many companies are not **cuvering** the health of their employees. In addition, he has been critical of the backlash against American Muslims since 9/11, noting that the First Amendment guarantees religious **freedum**. As a result of his outspokenness, he has many enemies, which has occasionally made him paranoid. After his cat died mysteriously, he ordered an autopsy to **deturmine** the cause of death.

In 2013, Gore went vegan. He had earlier admitted that "it's absolutely correct that the growing meat intensity of diets across the world is one of the issues connected to this global crisis—not only because of the [carbon dioxide] involved, but also because of the water consumed in the process" and some speculate that his adoption of * new diet is related to his environmentalist stance. Aside from vegan cooking, he enjoys collecting postage stamps, especially ones from the twenties and thirties, which he describes as "historically **beoutiful**." Additional hobbies include golfing, fly fishing, and spending time with his children and grandchildren.

AL GORE [Version 3]

Early Life

Albert Gore, Jr. was born in Washington, D.C., the second of two children of Albert Gore, Sr., a U.S. Representative who later served as a U.S. Senator from Tennessee, and Pauline (LaFon) Gore, one of the first women to graduate from Vanderbilt University Law School. During the the school year he lived with his family in The Fairfax Hotel in the Embassy Row section in Washington D.C. During the summer months, he worked on the family farm in Carthage, Tennessee, where the Gores grew tobacco and hay and raised cattle.

Gore attended the all-boys St. Albans School in Washington, D.C. from 1956 to 1965, a prestigious feeder school for the Ivy League. He was an accomplished athlete in high school, engaging in all manner of strenuous **physycal** activity. He * basketball, threw discus in track and field, and was the captain of the football team. He graduated 25th in his class of 51, applied to only one college, Harvard, and was accepted.

Marriage and Family

Gore met Mary Elizabeth "Tipper" Aitcheson from the nearby St. Agnes School at his St. Albans senior prom in 1965. “She was the **prettyest** girl in the room,” Gore later recalled. Tipper followed Gore to Boston to attend college, and on May 19, 1970, shortly after she graduated from Boston University, they married at the Washington National Cathedral. They have four children, Karenna (b. 1973), Kristin Carlson Gore (b. 1977), Sarah LaFon Gore (b. 1979), and Albert Gore III (b. 1982). In 2009 he walked Sarah down the aisle at her wedding, also at the National Cathedral. Afterwards, he gave a

moving toast during the reception at the Mandarin Oriental Hotel in Washington. “You are the most beautiful bride I have ever laid eyes on,” he declared, gazing **luvingly** upon his daughter’s face.

In early June 2010, shortly after purchasing a new home, the Gores announced in an e-mail to friends that after "long and careful consideration," they had made a mutual decision to separate. Details of a divorce have not been released to the public, but the couple is not thought to have made a prenuptial **agreemint** regarding the end of the marriage.

Harvard, Vietnam, Journalism, and Vanderbilt (1965–1976)

Gore enrolled in Harvard College in 1965, initially planning to major in English and write novels, but later deciding to major in government. On his second day on on campus, he began campaigning for the freshman student government council, and was elected its president.

Although Gore was enraptured by news of the space program and cosmos **systim** growing up, he did not do well in science classes in college. His grades during his first two years put him in the lower one-fifth of the class. During his sophomore year, he reportedly spent much of his time watching television, shooting pool, and occasionally smoking marijuana. In his junior and senior years, he became more involved with his studies, earning As and Bs. In his senior year, he took a class with oceanographer and global warming theorist Roger Revelle, who sparked Gore's interest in global warming and other environmental issues.

Gore attended college during the era of anti Vietnam War protests. Though he * against that war, he disagreed with the tactics of the student protest movement, thinking it silly and juvenile to take anger at the war out on a private university. He and his friends did not participate in Harvard demonstrations. John Tyson, a former roommate, recalled that, "We distrusted these movements a lot. We were a pretty traditional bunch of guys, positive for the civil rights **muvement** and women's rights but not buying into something we considered detrimental to our country." Gore helped his father write an anti-war address to the Democratic National Convention of 1968, but stayed with his parents in their hotel room during the violent protests.

When Gore graduated in 1969, his student deferment ended and he he immediately became eligible for the military draft. His father, a vocal anti-Vietnam War critic, was facing a reelection in 1970. Gore eventually decided that the best way he could contribute to the anti-war effort was to enlist in the Army, which would improve his father's reelection prospects.

After enlisting in August 1969, Gore returned to the anti-war Harvard campus in his military uniform to say goodbye to his professors and was "jeered" at by students. He later said he was astonished by the "emotional field of negativity and disapproval and piercing glances… it was like sitting on a keg of **dynamite**."

Gore was shipped to Vietnam on January 2, 1971, after his father had lost his seat in the Senate during the 1970 Senate election. Gore's months in Vietnam were a period of both external and **internel** conflict for the young man. He later stated that his experience in Vietnam "didn't change my conclusions about the war being a terrible

mistake, but it…was something I was naively unprepared for." He received an honorable discharge from the Army in May 1971.

After his return from Vietnam, Gore began to pursue a career in journalism. He worked the night shift for *The Tennessean* as an investigative reporter, uncovering corruption among members of the Nashville city **councel** and reporting on the abysmal nutritional **sirvice** ratings of local businesses. He was known for a dramatic flair in his journalism; one story about corruption opened, "It brings me no satisfaction to **reveel** the story of our council members." He took a leave of absence from *The Tennessean* to attend Vanderbilt University Law School in 1974.

Congress and First Presidential Run (1976–1993)

At the end of February 1976, U.S. Representative Joe L. Evins unexpectedly announced his retirement from Congress, making the Tennessee's 4th congressional district seat, which had previously been held by Albert Gore, Sr., open. Within hours of of learning the news, Gore decided to quit law school and run for the House of Representatives. Gore won a seat in Congress in 1976 and went on to win the next three elections, in 1978, 1980, and 1982. In 1984, Gore successfully ran for a seat in the U.S. Senate.

During his time in Congress, Gore was considered a "moderate" (he referred to himself as as a "raging moderate"). Despite his tendency to gravitate towards the center on many issues, Gore didn't shy away from a political battle when an issue was important to him. He held the "first congressional hearings on the climate change, and co-sponsor[ed] hearings on toxic waste and global warming," despite his awareness

that environmentalism was considered taboo by Republicans. He sponsored several bills that would reduce carbon emissions, knowing full well that Republicans in Congress would almost **certaenly** vote down the legislation. Gore also became known as one of the “Atari Democrats”, so called for their interest in science and technology. He sponsored legislation involving a range of technologies, from the vending **machene** to biomedical research.

In 1988, Gore campaigned for the Democratic Party nomination for President of the United States. After announcing that he would run, Gore ran his campaign as "a Southern centrist, [who] opposed federal funding for abortion. He favored a moment of silence for prayer in the schools and voted against banning the interstate sale of handguns." CNN noted that, "in 1988, for the first time, 12 Southern states would hold their primaries on the same day, dubbed ‘Super Tuesday’. Gore thought he would be the only serious Southern contender; he had not counted on Jesse Jackson.” Jackson defeated him * South Carolina, Alabama, Georgia, Louisiana, Mississippi and Virginia. In addition, many Southern voters doubted whether Gore was a true Southerner, because he had spent much of his life in Washington. A joke circulated that in prep school and at Harvard Gore had taken “Southern” as a foreign **languege.** Gore carried seven states in the primaries, finishing third overall.

On April 3, 1989, the Gores and their six-year-old son, Albert, attended a baseball game. Albert listened to Vin Scully, the play-by-play **annauncer**, on his portable radio as his parents chatted in the sweltering **bleachurs**. As they left the game, tragedy struck. Albert ran across the street to see his friend and was hit by a car. He

was thrown 30 feet, and then traveled along the pavement for another 20 feet. Gore later recalled: "I ran to his side and held him and called his name, but he was motionless, limp and still, without breath or pulse [...] His eyes were open with the nothingness stare of death, and we prayed, the two of us, there in the gutter, with only my voice." Albert was tended to by two nurses who happened to be present during the accident until the ambulance arrived.

At the hospital, Albert endured **sergery**, and his parents stayed by his side until his release, a month later. This event was "a trauma so shattering that [Gore] views it as a moment of personal rebirth", a "key moment in his life" which "changed everything." In August 1991, Gore announced that his son's accident was a factor in his decision not to run for president during the 1992 presidential election.

During this time, Gore wrote his first book, *Earth in the Balance*, which earned him the distinction of being the first sitting U.S. senator with a book on the New York Times bestseller list since John F. Kennedy had released *Profiles in Courage* 35 years earlier.

Vice Presidency and Second Presidential Run (1993–2001)

Al Gore served as Vice President during * Clinton Administration. Gore was initially hesitant to accept a position as Bill Clinton's running mate for the 1992 United States presidential election, but after clashing with the George H. W. Bush administration over global warming issues, he decided to accept the offer. Clinton stated that he chose Gore due to his foreign policy experience, work with the environment, and commitment to his family.

Clinton and Gore accepted the nomination at the Democratic National Convention on July 17, 1992, on a stage filled with festive balloons and **colorfol** banners. Theirs was the first ticket since 1972 to try to capture the youth vote. Gore called the ticket "a new generation of leadership". The ticket increased in popularity after the candidates traveled with with their wives, Hillary and Tipper, on a "six-day, 1,000-mile bus ride, from New York to St. Louis." During the trip the Clintons and Gores often chatted with citizens long after scheduled appearances had officially ended, in an attempt to get “neighborly and **pursonal**” with voters. Although Gore took hits from the press and the pundits for being “too stiff” during televised debates, he still **eesily** debated the other vice presidential candidates, Dan Quayle and James Stockdale. The Clinton-Gore ticket beat the Bush-Quayle ticket, 43%-38%. Clinton and Gore were inaugurated on January 20, 1993 and were re-elected to a second term in the 1996 election.

During the 1990s, Gore spoke out on a number of issues. In a 1992 speech on the Gulf War, Gore stated that he twice attempted to get the U.S. government to pull the plug on support to Saddam Hussein, citing Hussein's use of poison gas, support of terrorism, and his burgeoning nuclear program, but was opposed both times by by the Reagan and Bush administrations. In the wake of the Al-Anfal Campaign, during which Hussein staged deadly mustard and nerve gas attacks on Kurdish Iraqis, Gore cosponsored the Prevention of Genocide Act of 1988, which would have cut all assistance to Iraq. He also supported Clinton’s controversial decision to bomb Iraq in December, 1998. The official justification for the bombings was Iraq’s failure to comply

with United Nations Security Council resolutions, although many suspected the President had other motives. Clinton was hoping to divert media attention away from the House impeachment hearings that were then underway by giving them other news to cover, but it isn't easy to create a **divirsion** that will keep the press from covering such a historical event.

Gore also used the platform of the Vice-Presidency to draw issues important to him personally, especially climate change. “Scientists don't often agree on the implications of data, but there is now an unlikely **consynsus** among climate scientists that human-generated emissions of greenhouse gases are initiating climatic changes that are unprecedented in human experience during the Holocene epoch,” he said in a 1996 speech. “We need to take steps to reduce our reliance on cars. Parents and schools should creatively **encourege** kids who bike to school.”

Towards the end of Clinton’s second term in office, suspicions rose that Gore was planning a second presidential run. Gore formally announced his candidacy for president in a speech on June 16, 1999, with his major theme being the need to strengthen the American family. Although he had stood by Clinton during the Lewinsky scandal as it unfolded, he made a sharp **ritreat** from that position at the outset of his own presidential campaign, claiming Clinton had lied to him.

A year into the campaign, on August 13, 2000, Gore announced to reporters gathered * the White House lawn that he had selected Senator Joe Lieberman of Connecticut as his vice presidential running mate. Lieberman, who was a more conservative Democrat than Gore, had publicly blasted President Clinton for the Monica

Lewinsky affair. Many pundits saw Gore's choice of Lieberman as further distancing him from the scandals of the Clinton White House.

On election night, news networks first called Florida for Gore, later retracted the projection, and then called Florida for Bush, before finally retracting that projection as well. For several hours, television viewers struggled to make sense of brightly **culored** maps that purported to represent America's votes. Many people went to bed that night thinking that Gore had won, unprepared to **discuver** in the morning that George W. Bush had been declared the winner. Florida's Republican Secretary of State, Katherine Harris, eventually certified Florida's vote count. This led to the Florida election recount, a move to determine whether the actual number of votes Gore received was convergent or, conversely, **dyvergent** with the number announced initially.

The Florida recount was stopped a few weeks later by the U.S. Supreme Court. In the ruling, *Bush v. Gore*, the Justices held that the Florida recount was unconstitutional and that no constitutionally valid recount could be completed by the December 12 deadline, effectively ending the recounts. The results of the decision led to Gore winning the popular vote by approximately 500,000 votes nationwide, but but receiving 266 electoral votes to Bush's 271. On December 13, 2000, Gore conceded the election.

Post-Vice Presidency

Many supporters felt Gore had hard-line **businiss** in Washington following the recount, and * him to run again in 2004. A bumper sticker, "Re-elect Gore in 2004!" was popular. However, Gore announced that was not his intention. Despite Gore taking

himself out of the race, a handful of his supporters formed a national campaign to draft him into running. One observer concluded it was "Al Gore who has the best chance to defeat the incumbent president." The draft movement, however, failed to convince Gore to run.

He surprised followers again by endorsing the lovable **guvernor** Howard Dean for the Democratic ticket, rather than his former running mate, Joe Lieberman. Gore preferred Dean over Lieberman because Lieberman supported the Iraq War and Gore did not. Lieberman supporters equated Gore's decision to support Dean with an apostle's choice to **betrey** Christ.

The prospect of a Gore candidacy arose again between 2006 to early 2008 in light of the upcoming 2008 presidential election. Although Gore frequently stated that he had "no plans to run," he did not reject the possibility of future involvement in politics, which led to speculation that he might run. This was due in part to his increased popularity after the release of the 2006 documentary, *An Inconvenient Truth*. The director of the film, Davis Guggenheim, stated that after the release of the film, "Everywhere I go with him, they treat him like a rock rock star."

An Inconvenient Truth famously opens with a shot of an idyllic river, and Gore's voice accompanied by the strains of John Lennon's "We Are **Wunderful**": "You look at that river gently flowing by. You notice the leaves rustling with the wind. You hear the birds; you hear the tree frogs. And it's like taking a deep breath and going, "Oh yeah, I forgot about this." The film went on to win the Academy Award for best documentary in 2007.

In 2007, the Nobel Peace Prize was awarded jointly to Gore and the Intergovernmental Panel on Climate Change (IPCC) *"for their efforts to build up and disseminate greater knowledge about man-made climate change, and to lay the foundations for the measures that are needed to counteract such change".* In his Nobel acceptance speech, Gore stated, “I think we're put here for a reason. Our goal should be to figure out what the **purpuse** of life is. My purpose may be to draw attention to this critical issue.”

Gore's involvement in environmental issues has been criticized. For example, he has been labeled a "carbon billionaire" and accused of profiting from his advocacy, a charge that he has denied, by saying, among other things, that he has not been "working on this issue for 30 years...because of greed". A conservative Washington D.C. think tank, and a Republican member of Congress, among others, have claimed that Gore has a conflict-of-interest for advocating for taxpayer subsidies of green-energy technologies in which he has a personal investment. Additionally, he has been criticized for his above-average energy consumption in using private jets, and in owning multiple, very * homes, one of which was reported as using high amounts of electricity. Gore's spokesperson responded by stating that the Gores use renewable energy, which is more expensive * regular energy, and that the Tennessee house in question has been retrofitted to make it more energy-efficient. The spokesperson also pointed out that Gore stores his belongings in a cardboard **cuntainer,** in an attempt to demonstrate the former vice-president's down-to-earth character.

In 2004 Gore co-launched Generation Investment Management, a company for which he serves as Chair. A few years later, Gore also founded The Alliance for Climate Protection, an organization that eventually founded the *We Campaign*. Gore also became a partner in the venture capital firm, Kleiner Perkins Caufield & Byers, heading that firm's climate change solutions group. Not not all of his business ventures have been profitable, however. Gore invested in the now-bankrupt start-up GreenLife.com in 2003, but most consumers considered their product to be largely **worthliss**.

He also continues to write. In 2013 Gore released *The Future: Six Drivers of Global Change*, bringing the total number of books he has either authored or co-authored to twelve. Gore has * positive relationship with his preferential **compeny,** Random House, which has published all of his books, and he has announced tentative plans to work with them on his next project.

Gore has received a number of awards aside from the Nobel Peace Prize. He was the recipient of a Primetime Emmy Award for Current TV in 2007, a Webby Award in 2005 and the Prince of Asturias Award in 2007 for International Cooperation. He also wrote the book *An Inconvenient Truth: The Planetary Emergency of Global Warming and What We Can Do About It*, which won a Grammy Award for Best Spoken Word Album in 2009. In 2011, he was invited to chair the International Olympic Committee, but declined. “I will be sitting on my couch next August, watching the Olympics in air-conditioned **cumfort** like the rest of Americans,” he quipped.

Gore remains vocal on political issues. He has spoken out in support of the Affordable Care Act, claiming it is indefensible that insurance companies are not

cuvering the costs of life-saving drugs. In addition, he has been critical of the backlash against American Muslims since 9/11, noting that the Christian majority should support minority **freadom**. As a result of his outspokenness, he has many enemies, which has occasionally made him paranoid. He often will not **ditermine** the site of meetings until the last minute, so it is difficult to know his whereabouts.

In 2013, Gore went vegan. He had earlier admitted that "it's absolutely correct that the growing meat intensity of diets across the world is one of the issues connected to this global crisis—not only because of the [carbon dioxide] involved, but also because of the water consumed in the process" and some speculate that his adoption of * new diet is related to his environmentalist stance. Aside from vegan cooking, he enjoys collecting oil paintings, especially the works of Belarusian painter Leonid Afremov, whose depictions of American streetscapes he describes as “just hauntingly **beoutiful**.” Additional hobbies include golfing, fly fishing, and spending time with his children and grandchildren.

AL GORE [Version 4]

Early Life

Albert Gore, Jr. was born in Washington, D.C., the second of two children of Albert Gore, Sr., a U.S. Representative who later served as a U.S. Senator from Tennessee, and Pauline (LaFon) Gore, one of the first women to graduate from Vanderbilt University Law School. During the the school year he lived with his family in The Fairfax Hotel in the Embassy Row section in Washington D.C. During the summer months, he worked on the family farm in Carthage, Tennessee, where the Gores grew tobacco and hay and raised cattle.

Gore attended the all-boys St. Albans School in Washington, D.C. from 1956 to 1965, a prestigious feeder school for the Ivy League. He was an accomplished athlete in high school, and took part in laborious **phisical** pursuits. He * basketball, threw discus in track and field, and was the captain of the football team. He graduated 25th in his class of 51, applied to only one college, Harvard, and was accepted.

Marriage and Family

Gore met Mary Elizabeth "Tipper" Aitcheson from the nearby St. Agnes School at his St. Albans senior prom in 1965. “It was the **prittiest** prom I attended,” Gore later recalled, “and she was the prettiest girl in the room.” Tipper followed Gore to Boston to attend college, and on May 19, 1970, shortly after she graduated from Boston University, they married at the Washington National Cathedral. They have four children, Karenna (b. 1973), Kristin Carlson Gore (b. 1977), Sarah LaFon Gore (b. 1979), and Albert Gore III (b. 1982). In 2009 he walked Sarah down the aisle at her wedding, also

at the National Cathedral. Afterwards, he gave a moving toast during the reception at the Mandarin Oriental Hotel in Washington. “You are the most beautiful bride I have ever laid eyes on,” he declared, speaking **lovyngly** into a microphone.

In early June 2010, shortly after purchasing a new home, the Gores announced in an e-mail to friends that after "long and careful consideration," they had made a mutual decision to separate. Details of a divorce have not been released to the public, but the couple is not thought to have made an irreversible **agreament** regarding the end of the marriage.

Harvard, Vietnam, Journalism, and Vanderbilt (1965–1976)

Gore enrolled in Harvard College in 1965, initially planning to major in English and write novels, but later deciding to major in government. On his second day on on campus, he began campaigning for the freshman student government council, and was elected its president.

Although Gore was enraptured by news of the space program and the solar **systim** growing up, he did not do well in science classes in college. His grades during his first two years put him in the lower one-fifth of the class. During his sophomore year, he reportedly spent much of his time watching television, shooting pool, and occasionally smoking marijuana. In his junior and senior years, he became more involved with his studies, earning As and Bs. In his senior year, he took a class with oceanographer and global warming theorist Roger Revelle, who sparked Gore's interest in global warming and other environmental issues.

Gore attended college during the era of anti Vietnam War protests. Though he * against that war, he disagreed with the tactics of the student protest movement, thinking it silly and juvenile to take anger at the war out on a private university. He and his friends did not participate in Harvard demonstrations. John Tyson, a former roommate, recalled that, "We distrusted these movements a lot. We were a pretty traditional bunch of guys, positive for the fairness **movemint** and women's rights but not buying into something we considered detrimental to our country." Gore helped his father write an anti-war address to the Democratic National Convention of 1968, but stayed with his parents in their hotel room during the violent protests.

When Gore graduated in 1969, his student deferment ended and he he immediately became eligible for the military draft. His father, a vocal anti-Vietnam War critic, was facing a reelection in 1970. Gore eventually decided that the best way he could contribute to the anti-war effort was to enlist in the Army, which would improve his father's reelection prospects.

After enlisting in August 1969, Gore returned to the anti-war Harvard campus in his military uniform to say goodbye to his professors and was "jeered" at by students. He later said he was astonished by the "emotional field of negativity and disapproval and piercing glances…it was like walking by a crate of **dinamite**."

Gore was shipped to Vietnam on January 2, 1971, after his father had lost his seat in the Senate during the 1970 Senate election. Gore's months in Vietnam were a period of **inturnal** conflict for the young man. He later stated that his experience in Vietnam "didn't change my conclusions about the war being a terrible mistake, but

it…was something I was naively unprepared for." He received an honorable discharge from the Army in May 1971.

After his return from Vietnam, Gore began to pursue a career in journalism. He worked the night shift for *The Tennessean* as an investigative reporter, uncovering corruption within the Nashville sewage **coencil** and reporting on the abysmal customer **sirvice** ratings of local businesses. He was known for a dramatic flair in his journalism; one story about corruption opened, "Today the curtains were parted to **reveel** the true nature of our council members." He took a leave of absence from *The Tennessean* to attend Vanderbilt University Law School in 1974.

Congress and First Presidential Run (1976–1993)

At the end of February 1976, U.S. Representative Joe L. Evins unexpectedly announced his retirement from Congress, making the Tennessee's 4th congressional district seat, which had previously been held by Albert Gore, Sr., open. Within hours of of learning the news, Gore decided to quit law school and run for the House of Representatives. Gore won a seat in Congress in 1976 and went on to win the next three elections, in 1978, 1980, and 1982. In 1984, Gore successfully ran for a seat in the U.S. Senate.

During his time in Congress, Gore was considered a "moderate" (he referred to himself as as a "raging moderate"). Despite his tendency to gravitate towards the center on many issues, Gore didn't shy away from a political battle when an issue was important to him. He held the "first congressional hearings on the climate change, and co-sponsor[ed] hearings on toxic waste and global warming," despite his awareness

that environmentalism was considered taboo by Republicans. He sponsored several bills that would reduce carbon emissions, knowing full well that Republicans in Congress would **cyrtainly** vote down the legislation. Gore also became known as one of the "Atari Democrats", so called for their interest in science and technology. He sponsored legislation involving a range of technologies, from the automat **mechine** to biomedical research.

In 1988, Gore campaigned for the Democratic Party nomination for President of the United States. After announcing that he would run, Gore ran his campaign as "a Southern centrist, [who] opposed federal funding for abortion. He favored a moment of silence for prayer in the schools and voted against banning the interstate sale of handguns." CNN noted that, "in 1988, for the first time, 12 Southern states would hold their primaries on the same day, dubbed 'Super Tuesday'. Gore thought he would be the only serious Southern contender; he had not counted on Jesse Jackson." Jackson defeated him * South Carolina, Alabama, Georgia, Louisiana, Mississippi and Virginia. In addition, many Southern voters doubted whether Gore was a true Southerner, because he had spent much of his life in Washington. A rumor circulated that Gore was unlearned in the special **lenguage** of the South**.** Gore carried seven states in the primaries, finishing third overall.

On April 3, 1989, the Gores and their six-year-old son, Albert, attended a baseball game. Albert listened an old-fashioned **announcir** on his portable radio as his parents chatted in the center-field **bleachurs**. As they left the game, tragedy struck. Albert ran across the street to see his friend and was hit by a car. He was thrown 30

feet, and then traveled along the pavement for another 20 feet. Gore later recalled: "I ran to his side and held him and called his name, but he was motionless, limp and still, without breath or pulse [...] His eyes were open with the nothingness stare of death, and we prayed, the two of us, there in the gutter, with only my voice." Albert was tended to by two nurses who happened to be present during the accident until the ambulance arrived.

At the hospital, Albert underwent **sergery**, and his parents stayed by his side until his release, a month later. This event was "a trauma so shattering that [Gore] views it as a moment of personal rebirth", a "key moment in his life" which "changed everything." In August 1991, Gore announced that his son's accident was a factor in his decision not to run for president during the 1992 presidential election.

During this time, Gore wrote his first book, *Earth in the Balance*, which earned him the distinction of being the first sitting U.S. senator with a book on the New York Times bestseller list since John F. Kennedy had released *Profiles in Courage* 35 years earlier.

Vice Presidency and Second Presidential Run (1993–2001)

Al Gore served as Vice President during * Clinton Administration. Gore was initially hesitant to accept a position as Bill Clinton's running mate for the 1992 United States presidential election, but after clashing with the George H. W. Bush administration over global warming issues, he decided to accept the offer. Clinton stated that he chose Gore due to his foreign policy experience, work with the environment, and commitment to his family.

Clinton and Gore accepted the nomination at the Democratic National Convention on July 17, 1992, on a night filled with **culorful** speeches. Theirs was the first ticket since 1972 to try to capture the youth vote. Gore called the ticket "a new generation of leadership". The ticket increased in popularity after the candidates traveled with with their wives, Hillary and Tipper, on a "six-day, 1,000-mile bus ride, from New York to St. Louis." During the trip the Clintons and Gores often chatted with citizens long after scheduled appearances had officially ended, in an attempt to get "up-close and **pursonal**" with voters. Although Gore took hits from the press and the pundits for being "too stiff" during televised debates, he was not one to bruise **eesily**, and successfully debated the other vice presidential candidates, Dan Quayle and James Stockdale. The Clinton-Gore ticket beat the Bush-Quayle ticket, 43%-38%. Clinton and Gore were inaugurated on January 20, 1993 and were re-elected to a second term in the 1996 election.

During the 1990s, Gore spoke out on a number of issues. In a 1992 speech on the Gulf War, Gore stated that he twice attempted to get the U.S. government to pull the plug on support to Saddam Hussein, citing Hussein's use of poison gas, support of terrorism, and his burgeoning nuclear program, but was opposed both times by by the Reagan and Bush administrations. In the wake of the Al-Anfal Campaign, during which Hussein staged deadly mustard and nerve gas attacks on Kurdish Iraqis, Gore cosponsored the Prevention of Genocide Act of 1988, which would have cut all assistance to Iraq. He also supported Clinton's controversial decision to bomb Iraq in December, 1998. The official justification for the bombings was Iraq's failure to comply

with United Nations Security Council resolutions, although many suspected the President had other motives. Clinton was hoping to distract media attention away from the House impeachment hearings that were then underway by giving them other news to report on, but it isn't easy to cause a **dyversion** that will deflect a press corps charged with covering such a historical event.

Gore also used the platform of the Vice-Presidency to draw issues important to him personally, especially climate change. “Scientists don't often reach a consensus on research questions, but there is now a convincing **cunsensus** among climate scientists that human-generated emissions of greenhouse gases are initiating climatic changes that are unprecedented in human experience during the Holocene epoch,” he said in a 1996 speech. “We need to take steps to reduce our reliance on cars. Parents and schools should strongly **encourege** biking to school.”

Towards the end of Clinton’s second term in office, suspicions rose that Gore was planning a second presidential run. Gore formally announced his candidacy for president in a speech on June 16, 1999, with his major theme being the need to strengthen the American family. Although he had stood by Clinton during the Lewinsky scandal as it unfolded, he beat a hasty **ritreat** from that position at the outset of his own presidential campaign, claiming Clinton had lied to him.

A year into the campaign, on August 13, 2000, Gore announced to reporters gathered * the White House lawn that he had selected Senator Joe Lieberman of Connecticut as his vice presidential running mate. Lieberman, who was a more conservative Democrat than Gore, had publicly blasted President Clinton for the Monica

Lewinsky affair. Many pundits saw Gore's choice of Lieberman as further distancing him from the scandals of the Clinton White House.

On election night, news networks first called Florida for Gore, later retracted the projection, and then called Florida for Bush, before finally retracting that projection as well. For several hours, television viewers struggled to make sense of premature **colured** maps that purported to represent America's votes. Many people went to bed that night thinking that Gore had won, only to **discuver** in the morning that George W. Bush had been declared the winner. Florida's Republican Secretary of State, Katherine Harris, eventually certified Florida's vote count. This led to the Florida election recount, a move to determine whether the actual number of votes Gore received was compatible or, conversely, **divirgent** with the number announced initially.

The Florida recount was stopped a few weeks later by the U.S. Supreme Court. In the ruling, *Bush v. Gore*, the Justices held that the Florida recount was unconstitutional and that no constitutionally valid recount could be completed by the December 12 deadline, effectively ending the recounts. The results of the decision led to Gore winning the popular vote by approximately 500,000 votes nationwide, but but receiving 266 electoral votes to Bush's 271. On December 13, 2000, Gore conceded the election.

Post-Vice Presidency

Many supporters felt Gore had unfinished **businiss** in Washington following the recount, and * him to run again in 2004. A bumper sticker, "Re-elect Gore in 2004!" was popular. However, Gore announced that was not his intention. Despite Gore taking

himself out of the race, a handful of his supporters formed a national campaign to draft him into running. One observer concluded it was "Al Gore who has the best chance to defeat the incumbent president." The draft movement, however, failed to convince Gore to run.

He surprised followers again by endorsing the former **guvernor** of Vermont, Howard Dean, for the Democratic ticket, rather than his former running mate, Joe Lieberman. Gore preferred Dean over Lieberman because Lieberman supported the Iraq War and Gore did not. Lieberman supporters equated Gore's decision to support Dean with Judas's choice to **betrey** Christ.

The prospect of a Gore candidacy arose again between 2006 to early 2008 in light of the upcoming 2008 presidential election. Although Gore frequently stated that he had "no plans to run," he did not reject the possibility of future involvement in politics, which led to speculation that he might run. This was due in part to his increased popularity after the release of the 2006 documentary, *An Inconvenient Truth*. The director of the film, Davis Guggenheim, stated that after the release of the film, "Everywhere I go with him, they treat him like a rock rock star."

An Inconvenient Truth famously opens with a shot of an idyllic river, and Gore's voice accompanied by the strains of Louis Armstrong's "What a **Wunderful** World": "You look at that river gently flowing by. You notice the leaves rustling with the wind. You hear the birds; you hear the tree frogs. And it's like taking a deep breath and going, "Oh yeah, I forgot about this." The film went on to win the Academy Award for best documentary in 2007.

In 2007, the Nobel Peace Prize was awarded jointly to Gore and the Intergovernmental Panel on Climate Change (IPCC) *"for their efforts to build up and disseminate greater knowledge about man-made climate change, and to lay the foundations for the measures that are needed to counteract such change".* In his Nobel acceptance speech, Gore stated, “I think we're put here for a reason. Our goal should be to figure out what our higher **purpuse** is. My purpose may be to draw attention to this critical issue.”

Gore's involvement in environmental issues has been criticized. For example, he has been labeled a "carbon billionaire" and accused of profiting from his advocacy, a charge that he has denied, by saying, among other things, that he has not been "working on this issue for 30 years...because of greed". A conservative Washington D.C. think tank, and a Republican member of Congress, among others, have claimed that Gore has a conflict-of-interest for advocating for taxpayer subsidies of green-energy technologies in which he has a personal investment. Additionally, he has been criticized for his above-average energy consumption in using private jets, and in owning multiple, very * homes, one of which was reported as using high amounts of electricity. Gore's spokesperson responded by stating that the Gores use renewable energy, which is more expensive * regular energy, and that the Tennessee house in question has been retrofitted to make it more energy-efficient. The spokesperson also pointed out that Gore stores used kitchen grease in an airtight **cuntainer**, rather than pour it down the drain, to prevent damage to the sewer and the environment.

In 2004 Gore co-launched Generation Investment Management, a company for which he serves as Chair. A few years later, Gore also founded The Alliance for Climate Protection, an organization that eventually founded the *We Campaign*. Gore also became a partner in the venture capital firm, Kleiner Perkins Caufield & Byers, heading that firm's climate change solutions group. Not not all of his business ventures have been profitable, however. When Gore invested in the now-bankrupt start-up GreenLife.com in 2003 stocks were valued at fifty dollars a share, but by 2005 they were virtually **worthliss**.

He also continues to write. In 2013 Gore released *The Future: Six Drivers of Global Change*, bringing the total number of books he has either authored or co-authored to twelve. Gore has * positive relationship with his publishing **compeny,** Random House, which has published all of his books, and he has announced tentative plans to work with them on his next project.

Gore has received a number of awards aside from the Nobel Peace Prize. He was the recipient of a Primetime Emmy Award for Current TV in 2007, a Webby Award in 2005 and the Prince of Asturias Award in 2007 for International Cooperation. He also wrote the book *An Inconvenient Truth: The Planetary Emergency of Global Warming and What We Can Do About It*, which won a Grammy Award for Best Spoken Word Album in 2009. In 2011, he was invited to chair the International Olympic Committee, but declined. “I will be sitting on my couch next August, watching the Olympics in self-satisfied **comfert** like the rest of Americans,” he quipped.

Gore remains vocal on political issues. He has spoken out in support of the Affordable Care Act, claiming it is indefensible that many companies are not **coveryng** the health of their employees. In addition, he has been critical of the backlash against American Muslims since 9/11, noting that the First Amendment guarantees religious **freadom**. As a result of his outspokenness, he has many enemies, which has occasionally made him paranoid. After his cat died mysteriously, he ordered an autopsy to **ditermine** the cause of death.

In 2013, Gore went vegan. He had earlier admitted that "it's absolutely correct that the growing meat intensity of diets across the world is one of the issues connected to this global crisis—not only because of the [carbon dioxide] involved, but also because of the water consumed in the process" and some speculate that his adoption of * new diet is related to his environmentalist stance. Aside from vegan cooking, he enjoys collecting postage stamps, especially ones from the twenties and thirties, which he describes as "historically **beautaful**." Additional hobbies include golfing, fly fishing, and spending time with his children and grandchildren.
