# Supplementary material for: Lexical Stress and Linguistic Predictability Influence Proofreading Behavior
Source: Front Psychol. 2016 Feb 9;7:96. doi: 10.3389/fpsyg.2016.00096 (PMC4746312; doi:10.3389/fpsyg.2016.00096)
Supplement: Supplementary file 3 [file Image_3.PDF]

APPENDIX C

Critical stimuli correctly spelled, in high-constraint and low-constraint contexts.

Critical Stimulus (CS)	Number of Times (out of ten) CS Supplied in Cloze Task	Length of Word Preceding CS	Passage Context
container	10	8	The spokesperson also pointed out that Gore stores used kitchen grease in an airtight **container,** rather than pour it down the drain, to prevent damage to the sewer and the environment.
	0	9	The spokesperson also pointed out that Gore stores his belongings in a cardboard **container,** in an attempt to demonstrate the former vice-president's down-to-earth character.
freedom	10	9	He has been critical of the backlash against American Muslims since 9/11, noting that the First Amendment guarantees religious **freedom**.
	0	8	He has been critical of the backlash against American Muslims since 9/11, noting that the Christian majority should support minority **freedom**.
colored	10	8	For several hours, television viewers struggled to make sense of brightly **colored** maps that purported to represent America's votes.
	0	9	For several hours, television viewers struggled to make sense of premature **colored** maps that purported to represent America's votes.
business	10	10	Many felt Gore had unfinished **business** in Washington following the recount, and expected him to run again in 2004.
	0	9	Many felt Gore had hard-line **business** in Washington following the recount, and expected him to run again in 2004.
personal	10	3	During the trip the Clintons and Gores often chatted with citizens long after scheduled appearances had officially ended, in an attempt to get "up-close and **personal** " with voters.
	0	3	During the trip the Clintons and Gores often chatted with citizens long after scheduled appearances had officially ended, in an attempt to get "neighborly and **personal** " with voters.
internal	10	3	Gore's months in Vietnam were a period of both external and **internal** conflict for the young man.
	1	2	Gore's months in Vietnam were a period of **internal** conflict for the young man.
machine	10	7	He sponsored legislation involving a range of technologies, from the vending **machine** to biomedical research.
	1	7	He sponsored legislation involving a range of technologies, from the automat **machine** to biomedical research.

Critical Stimulus (CS)	Number of Times (out of ten) CS Supplied in Cloze Task	Length of Word Preceding CS	Passage Context
agreement	9	10	Details of a divorce have not been released to the public, but the couple is not thought to have made a prenuptial **agreement** regarding the end of the marriage.
	0	12	Details of a divorce have not been released to the public, but the couple is not thought to have made an irreversible **agreement** regarding the end of the marriage.
prettiest	9	3	Gore met Mary Elizabeth "Tipper" Aitcheson from the nearby St. Agnes School at his St. Albans senior prom in 1965. "She was the **prettiest** girl in the room," Gore later recalled.
	0	3	Gore met Mary Elizabeth "Tipper" Aitcheson from the nearby St. Agnes School at his St. Albans senior prom in 1965. "It was the **prettiest** prom I attended," Gore later recalled.
determine	9	2	Gore has many enemies, which has occasionally made him paranoid. After his cat died mysteriously, he ordered an autopsy to **determine** the cause of death.
	0	3	Gore has many enemies, which has occasionally made him paranoid. He often will not **determine** the site of meetings until the last minute, so it is difficult to know his whereabouts.
reveal	9	2	He was known for a dramatic flair in his journalism. One story about corruption opened, *Today the curtains were parted to **reveal** the true nature of our council members.*
	0	2	He was known for a dramatic flair in his journalism. One story about corruption opened, *It brings me no satisfaction to **reveal** the story of our council members.*
governor	9	6	He surprised followers again by endorsing the former **governor** of the state of Vermont, Howard Dean, for the Democratic ticket, rather than his former running mate, Joe Lieberman.
	0	7	He surprised followers again by endorsing the lovable **governor** Howard Dean for the Democratic ticket, rather than his former running mate, Joe Lieberman.
language	9	7	A joke circulated that in prep school and at Harvard Gore had taken "Southern" as a foreign **language**.
	0	7	A rumor circulated that Gore was unlearned in the special **language** of the South.
announcer	9	12	Albert listened to Vin Scully, the play-by-play **announcer**, on his portable radio as his parents chatted in the center-field bleachers.
	1	13	Albert listened to an old-fashioned **announcer** on his portable radio as his parents chatted in the center-field bleachers.
movement	9	6	"We were a pretty traditional bunch of guys, positive for the civil rights **movement** and women's rights but not buying into something we considered detrimental to our country."
	1	8	"We were a pretty traditional bunch of guys, positive for the fairness **movement** and women's rights but not buying into something we considered detrimental to our country."
comfort	8	15	I will be sitting on my couch next August, watching the Olympics in air-conditioned **comfort** like the rest of Americans.
	0	14	I will be sitting on my couch next August, watching the Olympics in self-satisfied **comfort** like the rest of Americans.
company	8	10	Gore has a positive relationship with his publishing **company**, Random House, which has published

Critical Stimulus (CS)	Number of Times (out of ten) CS Supplied in Cloze Task	Length of Word Preceding CS	Passage Context
			all of his books, and he has announced tentative plans to work with them on his next project.
	0	12	Gore has a positive relationship with his preferential **company**, Random House, which has published all of his books, and he has announced tentative plans to work with them on his next project.
easily	8	6	Although Gore took hits from the press and the pundits for being "too stiff" during televised debates, he was not one to bruise **easily**, and successfully debated the other vice presidential candidates, Dan Quayle and James Stockdale.
	0	5	Although Gore took hits from the press and the pundits for being "too stiff" during televised debates, he still **easily** debated the other vice presidential candidates, Dan Quayle and James Stockdale.
lovingly	8	6	"And you are the most beautiful bride I have ever laid eyes on," he declared, gazing **lovingly** upon his daughter's face.
	0	8	"And you are the most beautiful bride I have ever laid eyes on," he declared, speaking **lovingly** into a microphone.
surgery	8	9	At the hospital, Albert underwent **surgery,** and his parents stayed by his side until his release, a month later.
	1	7	At the hospital, Albert endured **surgery,** and his parents stayed by his side until his release, a month later.
service	7	8	He worked the night shift for *The Tennessean* as an investigative reporter, uncovering corruption within members of the Nashville city council and reporting on the abysmal customer **service** ratings in the community.
	0	11	He worked the night shift for *The Tennessean* as an investigative reporter, uncovering corruption within members of the Nashville city council and reporting on the abysmal nutritional **service** ratings in the community.
system	7	5	Although Gore was enraptured by news of the space program and the solar **system** growing up, he did not do well in science classes in college.
	0	6	Although Gore was enraptured by news of the space program and cosmos **system** growing up, he did not do well in science classes in college.
wonderful	7	1	*An Inconvenient Truth* famously opens with a shot of an idyllic river, and Gore's voice accompanied by the strains of Louis Armstrong's "What a **Wonderful** World."
	0	3	*An Inconvenient Truth* famously opens with a shot of an idyllic river, and Gore's voice accompanied by the strains of John Lennon's "We Are **Wonderful**."
worthless	7	9	When Gore invested in the now-bankrupt start-up GreenLife.com in 2003 stocks were valued at fifty dollars a share, but by 2005 they were virtually **worthless**.
	0	7	Gore invested in the now-bankrupt start-up GreenLife.com in 2003, but most consumers considered their product to be largely **worthless**.
retreat	7	5	Although he had stood by Clinton during the Lewinsky scandal as it unfolded, he beat a hasty **retreat** from that position at the outset of his own presidential campaign, claiming Clinton had lied to him.

Critical Stimulus (CS)	Number of Times (out of ten) CS Supplied in Cloze Task	Length of Word Preceding CS	Passage Context
	0	5	Although he had stood by Clinton during the Lewinsky scandal as it unfolded, he made a sharp **retreat** from that position at the outset of his own presidential campaign, claiming Clinton had lied to him.
colorful	7	3	Clinton and Gore accepted the nomination at the Democratic National Convention on July 17, 1992, on a stage decorated with festive balloons and **colorful** banners.
	0	4	Clinton and Gore accepted the nomination at the Democratic National Convention on July 17, 1992, on a night filled with **colorful** speeches.
certainly	7	6	He sponsored several bills that would reduce carbon emissions, knowing full well that Republicans in Congress would almost **certainly** vote down the legislation.
	1	5	He sponsored several bills that would reduce carbon emissions, knowing full well that Republicans in Congress would **certainly** vote down the legislation.
betray	7	2	Lieberman supporters equated Gore's decision to support Dean with Judas's choice to **betray** Christ.
	1	2	Lieberman supporters equated Gore's decision to support Dean with an apostle's choice to **betray** Christ.
physical	7	9	He was an accomplished athlete in high school, engaging in all manner of strenuous **physical** activity.
	1	9	He was an accomplished athlete in high school, and took part in laborious **physical** pursuits.
purpose	7	6	I think we're put here for a reason. Our goal should be to figure out what our higher **purpose** is.
	1	3	I think we're put here for a reason. Our goal should be to figure out what the **purpose** of life is.
council	6	4	He worked the night shift for The Tennessean as an investigative reporter, uncovering corruption amongst members of the Nashville city **council** and reporting on the abysmal customer service ratings in the community.
	0	6	He worked the night shift for The Tennessean as an investigative reporter, uncovering corruption within the Nashville sewage **council** and reporting on the abysmal customer service ratings in the community.
divergent	6	9	This led to the Florida election recount, a move to determine whether the actual number of votes Gore received was convergent or, conversely, **divergent** with the number announced initially.
	0	9	This led to the Florida election recount, a move to determine whether the actual number of votes Gore received was compatible or, conversely, **divergent** with the number announced initially.
beautiful	6	10	Aside from vegan cooking, he enjoys collecting oil paintings, especially the works of Belarusian painter Leonid Afremov, whose depictions of American streetscapes he describes as "just hauntingly **beautiful**."
	0	12	Aside from vegan cooking, he enjoys collecting postage stamps, especially ones from the twenties and thirties, which he describes as "historically **beautiful**."
encourage	6	8	We need to take steps to reduce our reliance on cars. Parents and schools should strongly **encourage** biking to school.

**Critical Stimulus (CS)**	**Number of Times (out of ten) CS Supplied in Cloze Task**	**Length of Word Preceding CS**	**Passage Context**
	1	10	We need to take steps to reduce our reliance on cars. Parents and schools should creatively **encourage** kids who bike to school.
bleachers	5	12	Albert listened to Vin Scully, the play-by-play announcer, on his portable radio as his parents chatted in the center-field **bleachers**.
	0	10	Albert listened to Vin Scully, the play-by-play announcer, on his portable radio as his parents chatted in the sweltering **bleachers**.
dynamite	5	2	He later said he was astonished by the "emotional field of negativity and disapproval and piercing glances…it was like sitting on a keg of **dynamite**.”
	0	2	He later said he was astonished by the "emotional field of negativity and disapproval and piercing glances...it was like walking by a crate of **dynamite**.”
discover	5	2	Many people went to bed that night thinking that Gore had won, only to **discover** in the morning that George W. Bush had been declared the winner.
	1	2	Many people went to bed that night thinking that Gore had won, unprepared to **discover** in the morning that George W. Bush had been declared the winner.
covering	5	3	Gore has spoken out in support of the Affordable Care Act, claiming it is indefensible that insurance companies are not **covering** the costs of life-saving drugs.
	1	3	Gore has spoken out in support of the Affordable Care Act, claiming it is indefensible that many companies are not **covering** the health of their employees.
diversion	5	1	Clinton was hoping to divert media attention away from the House impeachment hearings that were then underway by giving them other news to cover, but it isn't easy to create a **diversion** that will keep the press from covering such a historical event.
	1	1	Clinton was hoping to distract media attention away from the House impeachment hearings that were then underway by giving them other news to report on, but it isn't easy to cause a **diversion** that will deflect a press corps charged with covering such a historical event.
consensus	5	8	Scientists don't often agree on the implications of data, but there is now an unlikely **consensus** among climate scientists that human-generated emissions of greenhouse gases are initiating climatic changes that are unprecedented in human experience during the Holocene epoch.
	1	10	Scientists don't often reach a consensus on research questions, but there is now a convincing **consensus** among climate scientists that human-generated emissions of greenhouse gases are initiating climatic changes that are unprecedented in human experience during the Holocene epoch.
