# Supplementary material for: Lexical Stress and Linguistic Predictability Influence Proofreading Behavior
Source: Front Psychol. 2016 Feb 9;7:96. doi: 10.3389/fpsyg.2016.00096 (PMC4746312; doi:10.3389/fpsyg.2016.00096)
Supplement: Supplementary file 4 [file Image_4.PDF]

APPENDIX D

Proofreading passage instructions, including practice paragraph and comprehension question.

Welcome!

In a moment, you will be asked to proofread the Wikipedia entry for Al Gore. Following the task, you will be asked a few comprehension questions about the passage.

You will be looking for three types of errors: *misspellings*, *repetitions*, and *omissions*. A repetition is a word that is printed twice in a row. An omission is a word that is missing from a sentence, with the result that the sentence no longer makes sense.

Please circle any *misspellings* and *repetitions,* and write an 'X' in the place of an *omission.*

Please read the following paragraph at a natural pace, and mark any errors that you notice. A comprehension question will follow.

Albert Arnold "Al" Gore, Jr. (born March 31, 1948) is an Amarican politician, advocate and philanthropist, who served as the 45th Vice President of the United States (1993–2001), under President Bill Clinton. He was the Democratic Party's nominee for President and lost the 2000 U.S. presidential election despite winning the populer vote. Gore currently an author and environmental activist. He has founded a number of non-profit organizations, including the Alliance for Climate Protection, and has has received a Nobel Peace Prize for his work in climate change activism.

Comprehension

Please answer without referring back to the paragraph.

1. Gore lost the 2000 presidential election despite winning the _______________ vote.

The above paragraph contains four errors:

- You should have circled the misspelled words *Amarican* and *populer.*
- You should also have circled *has* at the beginning of the last line, which is a repetition.
- Finally, you should have written an 'X' before or after *currently*, because the word 'is' has been omitted.

Note that most paragraphs in the Wikipedia entry will not contain as many errors as the above practice paragraph.

Also note that you **are not responsible** for detecting errors of **punctuation**, **capitalization**, or **grammar**.

Please read at a natural pace.

The exercise should take approximately 15-25 minutes to complete. An experimenter will be nearby throughout the experiment should you have any questions.

Good luck!
