# Supplementary material for: Lexical Stress and Linguistic Predictability Influence Proofreading Behavior
Source: Front Psychol. 2016 Feb 9;7:96. doi: 10.3389/fpsyg.2016.00096 (PMC4746312; doi:10.3389/fpsyg.2016.00096)
Supplement: Supplementary file 5 [file Image_5.PDF]

APPENDIX E

Comprehension and feedback questions that followed all four versions of the proofreading passage.

Comprehension

Please do not refer back to the passage when responding.

1. After college, Gore enlisted in the Army and briefly served in the conflict in

   ______________________________.

2. Gore served in Congress as a Representative and Senator from the state of

   ______________________________.

3. Gore's six-year-old son was hit by a car after attending a _________________________ game.

4. In 2007, the _________________________________ was awarded jointly to Gore and the Intergovernmental Panel on Climate Change.

Feedback

What was your impression of the style of writing used in the passage you proofread?

What do you think is the purpose of this experiment?
